# Clinical, gut microbial and neural effects of a probiotic add-on therapy in depressed patients: a randomized controlled trial

**DOI:** 10.1038/s41398-022-01977-z

**Published:** 2022-06-03

**Authors:** Anna-Chiara Schaub, Else Schneider, Jorge F. Vazquez-Castellanos, Nina Schweinfurth, Cedric Kettelhack, Jessica P. K. Doll, Gulnara Yamanbaeva, Laura Mählmann, Serge Brand, Christoph Beglinger, Stefan Borgwardt, Jeroen Raes, André Schmidt, Undine E. Lang

**Affiliations:** 1 University of Basel, Department of Psychiatry (UPK), Basel, Switzerland; 2 Department of Microbiology and Immunology, Rega Institute for Medical Research, KU Leuven-University of Leuven, Leuven, Belgium; 3 VIB Center for Microbiology, Leuven, Belgium; 4 Sleep Disorders Research Center, Kermanshah University of Medical Sciences, Kermanshah, 6719851115 Iran; 5 Substance Abuse Prevention Research Center, Kermanshah University of Medical Sciences, Kermanshah, 6715847141 Iran; 6 Department of Sport, Exercise and Health, Division of Sport Science and Psychosocial Health, University of Basel, 4052 Basel, Switzerland; 7 School of Medicine, Tehran University of Medical Sciences, Tehran, 1416753955 Iran; 8 Department of Research, St. Clara Hospital, Basel, Switzerland; 9 Department of Psychiatry and Psychotherapy, University of Lübeck, Lübeck, Germany

**Keywords:** Depression, Molecular neuroscience, Physiology

## Abstract

A promising new treatment approach for major depressive disorder (MDD) targets the microbiota-gut-brain (MGB) axis, which is linked to physiological and behavioral functions affected in MDD. This is the first randomized controlled trial to determine whether short-term, high-dose probiotic supplementation reduces depressive symptoms along with gut microbial and neural changes in depressed patients. Patients with current depressive episodes took either a multi-strain probiotic supplement or placebo over 31 days additionally to treatment-as-usual. Assessments took place before, immediately after and again four weeks after the intervention. The Hamilton Depression Rating Sale (HAM-D) was assessed as primary outcome. Quantitative microbiome profiling and neuroimaging was used to detect changes along the MGB axis. In the sample that completed the intervention (probiotics *N* = 21, placebo *N* = 26), HAM-D scores decreased over time and interactions between time and group indicated a stronger decrease in the probiotics relative to the placebo group. Probiotics maintained microbial diversity and increased the abundance of the genus *Lactobacillus*, indicating the effectivity of the probiotics to increase specific taxa. The increase of the *Lactobacillus* was associated with decreased depressive symptoms in the probiotics group. Finally, putamen activation in response to neutral faces was significantly decreased after the probiotic intervention. Our data imply that an add-on probiotic treatment ameliorates depressive symptoms (HAM-D) along with changes in the gut microbiota and brain, which highlights the role of the MGB axis in MDD and emphasizes the potential of microbiota-related treatment approaches as accessible, pragmatic, and non-stigmatizing therapies in MDD. Trial Registration: www.clinicaltrials.gov, identifier: NCT02957591.

## Introduction

Major depressive disorder (MDD) is one of the most prevalent and burdensome psychiatric disorders [1] but current treatment options are still unsatisfying. Two-thirds of depressed patients do not respond adequately to initial antidepressant medication [2] and up to 30% of treatment-resistant patients experience residual symptoms when receiving optimized treatments [3]. The development of novel and more efficient treatment approaches is therefore urgently needed. Compelling preclinical data indicate that the gut microbiota affects brain functions and depressive behavior [4], providing a promising novel target for the treatment of depression [5–7]. In support of this preclinical research, pioneering studies reported alterations in gut microbiota composition in patients with depression [8–12], and relationships between gut microbiota and quality of life and depression in a large population cohort [13]. Moreover, Fecal Microbiota Transplantation (FMT) of stool derived from MDD patients induced depression-like behaviors in mice [8, 10], indicating a causal role of gut microbiota in depression.

A recent meta-analysis has demonstrated the potential of probiotic treatments for ameliorating mild and moderate depressive symptoms in patients suffering from several illnesses [14]. However, empirical data in patients with MDD remain scarce. So far, there is evidence that a 90 days administration of *Bacillus coagulans* improved depressive symptoms in patients with a combined diagnosis of MDD and irritable bowel syndrome (IBS) [15]. Randomized controlled trials (RCT) further found improvement in self-reported depressive symptoms in patients with MDD after an eight-week probiotic supplementation [16, 17]. However, another recent meta-analysis [18] indicate that probiotics are effective in reducing depressive symptoms when administered in addition to antidepressants but not when used as stand-alone treatment. This claim is in line with preclinical research showing that antidepressants increased gut microbiota diversity and that certain bacteria such as *Ruminococcus flavefaciens* specifically abolished the antidepressant effect of duloxetine on depressive-like behavior in mice [19].

Only a limited number of studies have explored probiotic effects on gut microbiota and brain functions in participants with depressive symptoms including IBS patients. While some studies could not find any probiotic-induced changes in gut microbiota [20–22], others reported increased abundance of *Ruminococcus gauvreauii* [22], decreased abundance of *Bacteroides* [23] and increased microbial diversity measures such as evenness at genus level [23]. Neuroimaging studies in healthy subjects and IBS patients reported reduced activation in resting-state networks [24] and regions related to cognition [25] and emotion [21, 26]. Notably, reduced amygdala responses to fearful faces correlated with changes in depressive symptoms in IBS patients after the probiotic treatment [21], providing a first indication of possible neural mechanisms underlying the effect of probiotic treatment on depressive symptoms.

However, the effects of probiotic supplementation on symptoms, gut microbiota and brain markers have never been investigated jointly in MDD patients. In this RCT, we examined the effect of a short-term, high-dose probiotic add-on therapy on depressive symptoms in MDD patients. Moreover, and for the first time, we explored the effects of a probiotic supplementation on gut microbiota composition as well as brain structure and function. Compared to the placebo group, we hypothesized that probiotics would ameliorate depressive symptoms immediately after a 4-week intervention and that the effect would remain 4 weeks post-intervention. In accordance with previous studies in IBS patients [23, 27], we further hypothesized increased microbial diversity after probiotic supplementation. Given the recently reported association between *Prevotella* enterotype and positive emotional well-being as well as *Bacteroides* 2 enterotype and depression [13], we also expected a shift in bacterial community towards increased abundance of *Prevotella* and decreased abundance of *Bacteroides* 2 enterotypes after probiotic supplementation. Finally, in line with a previous IBS study [21], we predicted reduced amygdala activation in response to fearful faces after probiotic supplementation but no effect on brain structure due to the relatively short intervention period.

## Patients and methods

The study was a double-blind RCT of a probiotic add-on therapy for four weeks in depressed patients. Data were collected between March 2017 and January 2020 in Basel, Switzerland. The study was approved by the local ethics committee (Ethikkommission Nordwest- und Zentralschweiz) and conducted in accordance with the Declaration of Helsinki. It was registered at www.clinicaltrials.gov prior to the study start (identifier NCT02957591).

### Participants

Patients with current depressive episodes (F31.3-F34 according to ICD-10 criteria) were recruited in an inpatient setting at the University Psychiatric Clinics, Basel, Switzerland. Participants fulfilled the eligibility criteria such as Hamilton Rating Scale for Depression (HAM-D [28]) score >7 (mild depression) [29], age ≥18 years and treatment as usual (TAU) for depression. During recruitment, eligibility criteria were adapted to improve the recruitment rate since not enough patients fulfilled the initially defined criterion of a severe depression (HAM-D > 24). Patients had to be able to read and understand the participant’s information and give informed consent. Immunosuppressed patients and patients with dietary restrictions and medical conditions such as acute infectious diseases were excluded. Pregnancy, breast-feeding and comorbid psychiatric disorders such as addiction, bipolar disorder and schizophrenia were also exclusion criteria.

Sample size estimation was calculated for the primary endpoint HAM-D via G*Power [30]. Since, at that time, no data was available for HAM-D changes due to probiotic supplementations in depressed patients, we assumed a medium effect size (*f* = 0.25) which resulted in a total sample size of 44 subjects for a repeated-measures ANOVA with three time points ensuring a power (1-β) of 90% and a significance level of *p* = 0.05. To take into account the high dropout rate in RCTs with depressed patients [31, 32], we recruited 60 patients, which equals the estimated sample size plus 30% dropouts. This sample size is in line with previous studies investigating the effect of probiotics on clinical outcomes, typically having sample sizes of 40–75 in total [15, 17, 20, 21, 33–36].

### Study intervention

In addition to TAU, patients took a probiotic supplement (Vivomixx®, Mendes SA, Lugano, Switzerland) containing eight different strains (*Streptococcus thermophilus* NCIMB 30438*, Bifidobacterium breve* NCIMB 30441*, Bifidobacterium longum* NCIMB 30435 (Re-classified as *B. lactis), Bifidobacterium infantis* NCIMB 30436 (Re-classified as *B. lactis), Lactobacillus* acidophilus NCIMB 30442*, Lactobacillus plantarum* NCIMB 30437*, Lactobacillus paracasei* NCIMB 30439*, Lactobacillus delbrueckii subsp. Bulgaricus* NCIMB 30440 *(*Re-classified as *L. helveticus))*. The daily dose contained 900 billion CFU/day that could be mixed with any cold, non-carbonated drink. As there is still no clear evidence which specific bacteria improve depressive symptoms, we decided to use a probiotic supplement that it is easily accessible in drug stores and, thus, easy to implement in clinical practice. To the best of our knowledge, no previous study investigating effects on depressive symptoms used such a high dosage. In the control group, participants received a placebo containing maltose but no bacteria. The placebo was indistinguishable in color, shape, size, packaging, smell, and taste from the probiotic supplement. The intervention was supplied by nursing personnel and patients who quit the inpatient setting during the intervention were instructed to continue the intervention.

### Study design and procedure

Patients were randomly allocated to the two study groups and tested at three different time points before the intervention, directly after and again four weeks after the intervention (Supplementary Fig. 1). In the baseline assessment, patients completed a test battery consisting of demographics, clinical measures, brain imaging and stool sampling. The study intervention took four weeks (31 days) as previous studies could find effects after the same period [36, 37]. Afterwards, patients completed the same test battery as before. Four weeks after the end of the intervention, they completed a follow-up assessment without brain imaging. An eight weeks observation is recommended to declare whether described treatments in depression care are ineffective and should be altered [38]. During the intervention period, patients’ usual medication was registered (see Supplementary Methods and Supplementary Table 1). During inpatient setting, patients received a standardized diet containing stable amounts of fibers and starch.

### Clinical measures

The primary outcome of the study was the HAM-D. Additionally, German versions of the Beck Depression Inventory (BDI) [39], the Gastrointestinal Symptom Rating Scale (GSRS) [40] and the State-Trait Anxiety Inventory 1 (STAI1) [41] were used to assess self-reported depressive symptoms, gastro-intestinal symptoms and anxiety, respectively.

### Statistical analysis of clinical measures

We used mixed-effects models to predict HAM-D scores including time as within factor with three levels (baseline, post-intervention, follow-up), treatment group as between factor (probiotics, placebo) and a random intercept. An analysis of variance (ANOVA, type III) was run over the model. To avoid confounding effects, analyses of significant interactions were repeated including potential confounding variables (sex, age, BMI, medication). Post-hoc tests of significant interactions were calculated by comparing changes of symptoms over time (change scores) between groups. Between-group effect sizes (Cohen’s d) were computed based on those change scores and a model was conducted with HAM-D baseline adjustment. Secondary outcomes were analyzed in the same manner. All analyses were conducted with the intention-to-treat (ITT) sample including all participants and a modified intention-to-treat (mITT) sample including only compliant participants. For details and outcome transformations see Supplementary Methods.

### Gut microbiota

#### Microbiota data sequencing and processing

Stool samples were taken by the participants and stored at −80 °C until DNA extraction. Fecal DNA was extracted following the protocol described by Falony et al. [42]. In brief, DNA was extracted from 150–200 mg of the frozen samples and the V4 region of 16S ribosomal RNA (rRNA) genes was amplified, purified, and sequenced. The microbial load of the study cohort was measured by flow cytometry [43]. For 16S rRNA data processing, amplicon data from the 16S rRNA gene was analyzed following the DADA2 pipeline specifications [44]. Fecal moisture and calprotectin concentrations were determined. For specifications of microbiota sequencing, quantitative microbiome profiling, and processing steps see Supplementary Methods.

#### Enterotyping

The 16S rRNA bacterial profiles were collapsed at the genus level and integrated along with the Belgian Flemish Gut Flora Project (FGFP) cohort [42]. Identification of the enterotypes was accomplished with the Dirichlet-multinomial Model approach. A matched sample of 93 healthy subjects from the FGFP cohort [42] was included, and the amplicon sequences data were processed as described before (details see Supplementary Methods).

#### Diversity measures

As alpha-diversity measures, observed richness, Pielou’s evenness, Shannon and inverse Simpson indices were estimated for all samples. Beta diversity was estimated from the 16S rRNA amplicon sequence variant (ASV) data. The Bray-Curtis index was used to estimate dissimilarities between samples in the even sampling depth ASV table. Detailed information about alpha and beta diversity analysis, statistical analysis and data visualization are described in the Supplementary Methods.

#### Bacterial taxa and associations with clinical measures

To explore effects of the probiotic supplement on bacterial taxa over time, mixed models were conducted including both study groups and separately per group. Also, associations of clinical measures (HAM-D, BDI, GSRS, STAI1) with affected bacterial taxa were explored using mixed models. Relevant specifications are in the Supplementary Methods.

### Brain structure and function

To reveal structural brain changes due to the probiotic intervention, voxel-based morphometry was performed using the Computational Anatomy Toolbox [45]. Additionally, patients underwent a well-established 6-min task examining emotional face processing including neutral and fearful faces [46, 47] using functional magnetic resonance imaging. Functional imaging data were analyzed using the full factorial design provided by SPM12 (http://www.fil.ion.ucl.ac.uk/spm/). In addition to the study sample, imaging data of a healthy control sample was used to specify our results. For further details on image acquisition and data analysis, see Supplementary Methods.

## Results

### Clinical and behavioral measures

Out of 60 included participants, 47 completed the intervention (*N*_probiotics_=21, *N*_placebo_ = 26), representing a dropout rate of 30% in the probiotics group and 13% in the placebo group (CONSORT diagram, Supplementary Fig. 2). Between post-intervention and follow-up assessment, one additional participant per group withdrew from the study (see Supplementary Results). Group comparisons showed equal demographic characteristics and no differences in clinical measures at baseline except HAM-D scores of the mITT sample (Table 1). The compliance cut-off rate of >65% [48] for the mITT sample resulted in the exclusion of two patients per group. Overall mean compliance rate increased to 88% and remained equal between groups (Table 1).

**Table 1:** Demographics and clinical measures of both study groups at baseline.

	ITT sample (*N* = 47)			mITT sample (*N* = 43)		
	Probiotics (*N* = 21)	Placebo (*N* = 26)	Group comparison	Probiotics (*N* = 19)	Placebo (*N* = 24)	Group comparison
*Demographics*						
Females, *N* (%)	14 (67)	13 (50)	χ^2^(1)=0.73, *p* = 0.49	14 (74)	12 (50)	χ^2^(1)=1.60, *p* = 0.21
Age, mean (SD)	39.43 (11.45)	38.77 (10.32)	*W* = 278, *p* = 0.92	39.21 (11.53)	38.04 (10.24)	*W* = 238.5, *p* = 0.81
BMI, mean (SD)	23.50 (3.67)	24.88 (3.95)	*W* = 207, *p* = 0.23	23.83 (3.66)	25.13 (4.01)	*W* = 177, *p* = 0.30
Smoking, *N* (%)						
≥ 1/day	7 (33)	11 (42)	χ^2^(1)=0.27, *p* = 0.60	5 (26%)	10 (42%)	χ^2^(1)=0.87, *p* = 0.35
NA	3 (14)	5 (19)		3	5	
Hospitalization, N (%)						
1	8 (38)	12 (46)	*W* = 224.5, *p* = 0.45	7	12	*W* = 210.5, *p* = 0.32
2	4 (19)	5 (19)		4	4	
3	3 (14)	3 (12)		3	3	
4	0 (0)	1 (4)		0	1	
5	3 (14)	0 (0)		3	0	
> 6	0 (0)	1 (4)		0	1	
NA	3 (14)	4 (15)		2	3	
Education, *N* (%)						
Primary	7 (33)	4 (15)	*W* = 269.5, *p* = 0.88	6	4	*W* = 237, *p* = 0.63
Secondary	3 (14)	12 (46)		3	12	
Tertiary	11 (52)	9 (35)		10	7	
NA	0 (0)	1 (4)		0	0	
*Medication, mean DDD (SD)*						
Antidepressant equivalents	1.73 (1.30)	1.79 (1.09)	*W* = 253, *p* = 0.68	1.86 (1.30)	1.82 (1.12)	*W* = 227, *p* = 0.99
Antipsychotic equivalents	0.30 (0.68)	0.22 (0.30)	*W* = 278, *p* = 0.92	0.33 (0.71)	0.24 (0.31)	*W* = 241, *p* = 0.76
*Clinical measures, mean score (SD)*						
HAM-D	18.93 (4.78)	16.5 (4.04)	*W* = 363, *p* = 0.05	19.13 (4.89)	16.5 (4.18)	*W* = 311, *p* = 0.04
BDI	22.38 (7.54)	22.33 (10.17)	*W* = 257.5, *p* = 0.96	21.53 (7.59)	22.31 (9.94)	*W* = 218.5, *p* = 0.96
STAI1	49.75 (13.89)	52.36 (10.40)	*W* = 229.5, *p* = 0.65	49 (14.11)	51.83 (10.61)	W = 191, *p* = 0.68
GSRS	28.52 (9.48)	29.83 (12.45)	*W* = 261, *p* = 0.98	28.16 (9.65)	29.96 (12.79)	W = 211.5, *p* = 0.87
Compliance, mean %, (SD)	83 (17.21)	86 (11.72)	*W* = 231, *p* = 0.76	87 (8.44)	88 (8.17)	W = 186, *p* = 0.84

*ITT* intention-to-treat, *mITT* modified intention-to-treat, *BMI* body mass index, *DDD* defined daily dose, *HAM-D* Hamilton Rating Scale for Depression, *BDI* Beck Depression Inventory, *STAI1* State-Trait Anxiety Inventory 1, GSRS=Gastrointestinal Symptom Rating Scale.

#### Probiotics improve depressive symptoms stronger than placebo

**Fig. 1:**
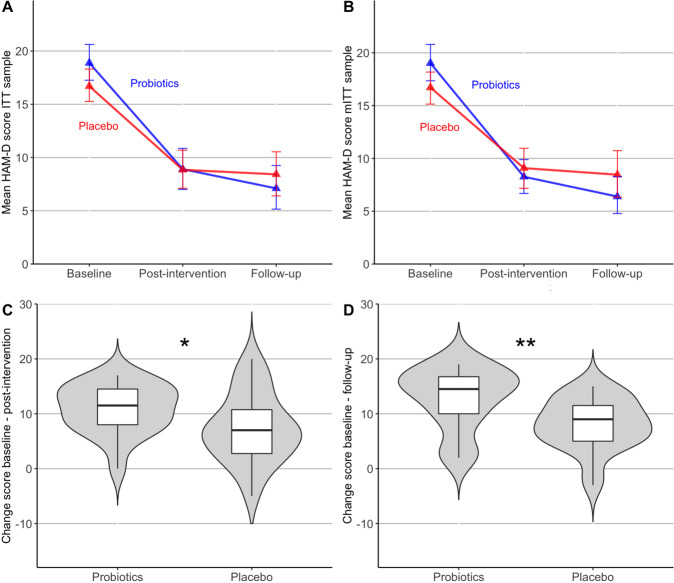
Trajectories and change scores of depressive symptoms in the probiotics and placebo group. Mean trajectory of scores on the Hamilton Scale for Depression (HAM-D) from baseline to post-intervention (week 4) and follow-up assessment (week 8) in **A** the intention-to-treat sample (ITT) and **B** the modified intention-to-treat sample (mITT). Error bars indicate 95% confidence interval (based on a bootstrap). **C** Change scores from baseline to post-intervention in the mITT sample and **D** from baseline to follow-up.

Mean trajectories showed a decrease of HAM-D scores over time and interactions between time and group indicated a stronger decrease in the probiotics group (Fig. 1A, B, Supplementary Fig. 3). A main effect of time was present in both the ITT (F(2, 99.69)=98.28, *p* < .001) and the mTT sample (F(2, 91.55)=100.56, *p* < .001), but the time*group interaction was only significant in the mITT sample (*F*(2, 91.55)=3.4, *p* < .05, Supplementary Table 2), which remained significant when controlling for confounders (*F*(2, 86.28)=3.74, *p* < .05). Change score comparisons showed a significant stronger decrease of HAM-D scores in the probiotics compared to the placebo group from baseline to post-intervention (t(40.55)=2.11, *p* < 0.05, *d* = 0.62) and from baseline to follow-up (*t*(32.98)=2.95, *p* < .01, *d* = 0.95) (Fig. 1C, D). After adjusting for HAM-D baseline scores, both post-hoc group effects remained significant (*F*(1)=6.66, *p* < 0.05, *F*(1)=12.65, *p* < 0.01). Treatment response (HAM-D change >57% [49]) at follow-up occurred in 80% of the patients in the probiotics and in 48% of the placebo group, indicating a strong statistical trend (χ^2^(1,45)=3.57, *p* = 0.06).

Analyses of secondary clinical measures showed significant symptoms decreases over time in the BDI, STAI1 and GSRS scores but no time*group interactions (Supplementary Tables 3–5). Change scores are presented in the Supplementary Results (ITT: Supplementary Fig. 4A–H, mITT: Supplementary Fig. 5A–F).

### Gut microbiota

Overall, 102 stool samples were available from all three time points, but after filtering on cell count data acquisition and sufficient sampling depth, a total of 89 samples from the mITT sample were analyzed. The study groups did not show any group differences in moisture, cell counts or calprotectin (Supplementary Table 6, Supplementary Fig. 6). The microbiome composition at baseline was dominated by species form the genera *Feacalibacterium, Roseburia, Bacteroides* and *Blautia* (Supplementary Fig. 7) and the overall enterotype distribution was driven by the abundance of Bacteroides 1 (31,18%) and Bacteroides 2 (31,18%) enterotypes. Further details and comparisons to healthy controls are in the Supplementary Results.

### Significant interaction of enterotype distribution

**Fig. 2:**
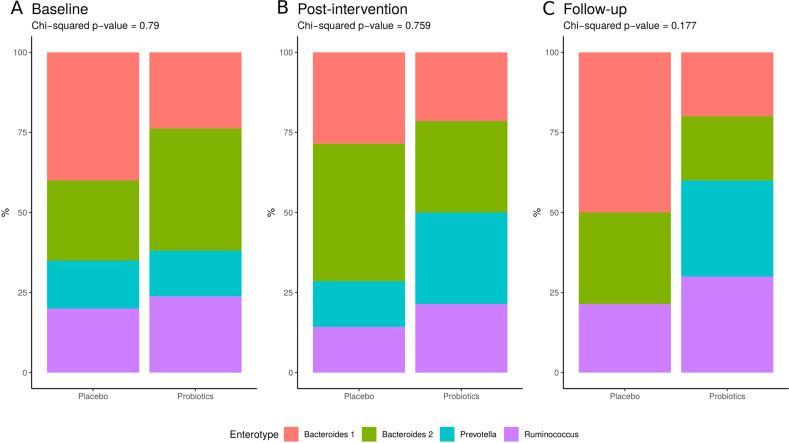
Enterotype distribution within time points. Group differences at baseline (**A**), post-intervention (**B**), and follow-up (**C**) per study groups.

There was a significant time*group interaction for the *Prevotella* (*F* = 4E−09, χ2 = 11.87, *p*-BH = 0.037) and *Rumminococcus* (*F* = 0.089, χ^2^ = 12.73, *p*-BH = 0.026) enterotypes, reflecting a loss of the *Prevotella* enterotype and an increase of the *Rumminococcus* enterotype at follow-up in the placebo group (Fig. 2A–C). However, there was neither a change in the overall enterotype composition over time within the study groups (Supplementary Fig. 10) (*p*-BH > 0.1), nor between the groups across time points (*p*-BH > 0.1).

### Probiotics maintain microbial diversity and overall community composition

**Fig. 3:**
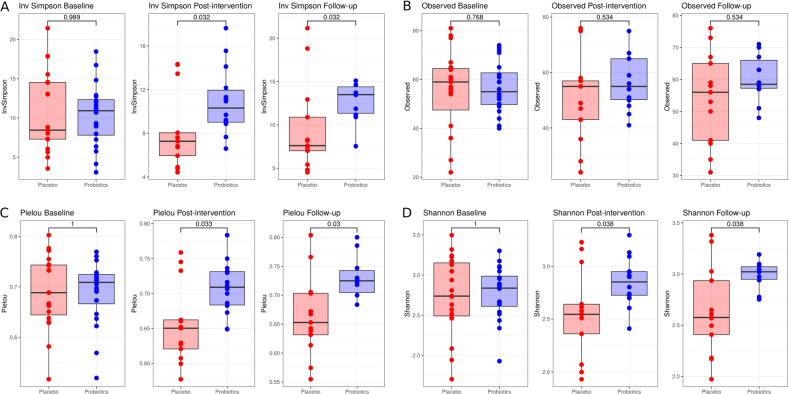
Alpha-diversity comparisons between study groups at all three time points. **A** Inversed Simpson index, **B** observed richness, **C** Pielou’s evenness, and **D** Shannon index.

Alpha-diversity measures showed no significant changes over time (Supplementary Fig. 11), neither in the probiotics group nor in placebo. However, when comparing the two study groups at post-intervention and follow-up, probiotics maintained diversity while the placebo group was reduced in inversed Simpson (Fig. 3A), Pielou’s evenness (Fig. 3C) and Shannon index (Fig. 3D) but not in observed richness (Fig. 3B).

Beta diversity results showed significant differences between study groups, moisture, subject, sex, BMI, and age (Supplementary Table 7, Supplementary Fig. 12A). However, after the stepwise confounding analysis only subject and moisture remained as non-redundant variables explaining 84% of the microbiome variation (Supplementary Fig. 12B). The analysis was repeated per time point; however, the difference between groups and the time*group interaction were not significant (Supplementary Table 7).

### Probiotics affect abundance of specific bacterial taxa

**Fig. 4:**
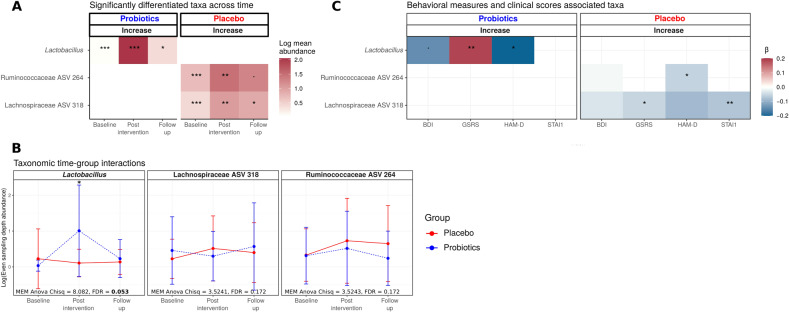
Significantly associated taxa to time and behavioral measures. (**A**) Mean abundance of the taxa showing a significant increase over the study intervention in the placebo and probiotic group, respectively (MEM ANOVA p-FDR < 0.05 and Wald test *p*-FDR < 0.05). ^•^<0.1, *<.05, **<0.01, *^*^*<0.001 *p*-FDR values after being adjusted for fecal moisture, sex, BMI, and age. Increases are indicated in relation to baseline values. (**B**) Line plots of the taxa-abundance of those taxa whose abundance differed significantly over time in the two study groups. The bottom of the panel shows ANOVA results for the time*group interaction, *p < .05 Wald test adjusted. (**C**) Mixed-effect β coefficients of the significantly associated taxa with the clinical measures (HAM-D, BDI, GSRS, STAI1) over time. The significance of the taxa was determined by using a negative binomial mixed-effect model; ^•^<0.1, *<0.05, **<0.01 ANOVA adjusted p-values after being adjusted for fecal moisture, sex, BMI, and age. The gradient color indicates the negative binomial mixed-effect model coefficient. HAM-D = Hamilton Rating Scale for Depression; BDI = Beck Depression Inventory; STAI1 = State-Trait Anxiety Inventory 1; GSRS = Gastrointestinal Symptom Rating Scale.

Subjects that received the probiotic supplement increased the abundance of the genus *Lactobacillus* after the intervention (Fig. 4A). This increase was not observed in the placebo group, in which ASVs of the family Ruminococcaceae and the family Lachnospiraceae showed an increase over time (Fig. 4A). In models including both study groups, the time*group interaction was only significant for the genus *Lactobacillus* with significant enrichment in the probiotics (Fig. 4B and Supplementary Fig. 13) compared to the placebo group. Contrary, the Ruminococcaceae and Lachnospiraceae ASVs did not change differently between the study groups (Fig. 4B). Interestingly, the Lachnospiraceae ASV strain showed a slight increase in the probiotics group at follow-up which was not significant (Fig. 4B).

### Associations of gut microbiota and clinical measures

In the probiotics group, the increase in abundance of the *Lactobacillus* genus showed a negative association with the HAM-D and BDI (Fig. 4C). In contrast, it showed a significant positive association with the GSRS. However, as indicated with the time effect, the GSRS was decreased over time. In the placebo group, the Ruminococcaceae ASV was associated with the decrease of the HAM-D. The Lachnospiraceae ASV was negatively associated with the GSRS and STAI1.

### Brain imaging

#### Probiotics increase gray matter volume in calcarine sulcus

We did not find any significant time*group interaction in the grey matter volume. However, the probiotics group showed increased grey matter volume in the calcarine sulcus extending in the lingual gyrus (MNI: *x* = 10, *y* = −81, *z* = 4; *k* = 950; *T*_max_ = 4.68; *p*_cluster(FWE)_ = 0.027) after the intervention compared to placebo. For the inverse contrast (placebo > probiotics group) and the respective contrasts at baseline, we did not observe any suprathreshold differences in grey matter volume. Additional analyses of thickness, gyrification and sulcus depth did not reveal any structural changes between groups over time (Supplementary Tables 8–10).

#### Probiotics alter putamen’s activation during emotion processing

**Fig. 5:**
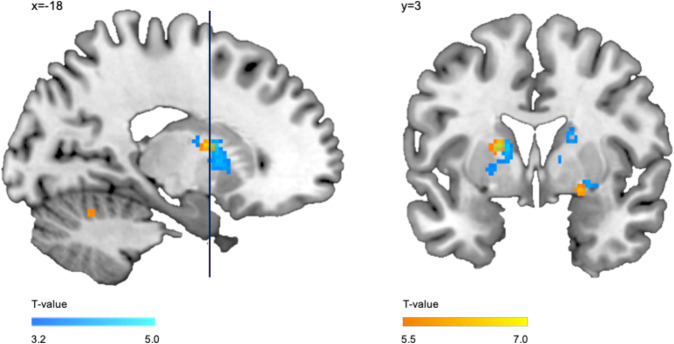
fMRI results: activation pattern during neutral face processing. Blue: decreased activation after the 4-week intervention in the probiotics group during neutral face processing. Red: increased activation in depressive patients (i.e. probiotics and placebo groups combined at baseline) compared to healthy controls during neutral face processing.

Examining activation changes over time, we found the two biggest clusters of all significant activation changes in the probiotics group during neutral face processing. There was a significant activation decrease in the right and left putamen after the probiotic intervention (right: *x* = 20, *y* = 16, *z* = 10; *k* = 251, *T*_max_ = 4.78, *p*_cluster(FWE)_ < 0.001 and left: *x* = −18, *y* = 6, *z* = 12; *k* = 223, *T*_max_ = 4.06, *p*_cluster(FWE)_ < 0.001; Fig. 5, Supplementary Table 11). We did not find any activation changes in these regions for the placebo group (Supplementary Table 12). Contrasting the probiotic against placebo group during neutral face processing, did, however, not reveal any significant differences between the two groups. Nevertheless, we found a significantly higher activation in these regions in depressive patients at baseline, i.e. probiotics and placebo groups together, compared to a sample of healthy controls (Fig. 5), indicating that the putamen is relevant in depression. Next to other regions, we observed a hyperactivation in the right amygdala extending into the right putamen and in the left putamen extending into the caudate nucleus in depressive patients compared to healthy controls during neutral faces processing (right: *x* = 22, *y* = 4, *z* = −10; *k* = 21; *T* = 6.31, *p*_peak(FWE)_ < .001, *p*_cluster(FWE)_ < 0.001 and left: *x* = −18, *y* = 2, *z* = 12; *k* = 31, *T* = 7.07; *p*_peak(FWE)_ < .001, *p*_cluster(FWE)_ < .001; Supplementary Table 13). For the results of semi-fearful (50%) and fearful (100%) faces see Supplementary Results for each study group (Supplementary Tables 11 and 12) and for depressive patients versus healthy controls (Supplementary Tables 14, 15).

We did not find any significant correlations between functional (or structural) changes and changes in clinical or microbiome measures.

## Discussion

In this RCT, we tested the potential of a short-term, high-dose probiotic supplementation as an add-on treatment for depression. Our main finding of a stronger amelioration of depressive symptoms after probiotic supplementation supports a previous study reporting a continued beneficial effect of probiotics on depressive symptoms but not anxiety in IBS patients [21]. Notably, in our study probiotic effects were only significant in a subsample with high compliance and accentuated in the follow-up after eight weeks, indicating a remission rate of 55% in the probiotics group compared to a 40% remission rate in the placebo group. The importance of compliance during probiotic supplementation should be highlighted and is as important as in general antidepressant therapy [50]. Heterogenic medication including polypharmacy of the patients was present due to their symptom severity and the inpatient setting. Despite this polypharmacy, the probiotic supplementation had a beneficial effect on the clinical progression. Interestingly, another study could only find a probiotic effect at the end of an eight-week intervention in treatment-resistant MDD patients but the effect did not persist at the 16-weeks follow-up [51]. However, this study included only 12 patients, was not placebo-controlled and the probiotic supplement contained different strains in lower dose than ours. Beneficial effects of probiotics on other clinical measures like self-reported depressive symptoms were not found in our study. Discrepancies in self-report vs. expert-rated depressive symptoms might be explained with the dependency of BDI self-report ratings on personality characteristics of the patient [52]. Although, it has been shown that the HAM-D is more sensitive to symptom change than the BDI [53], another study using only BDI self-report symptoms found a probiotics effects after an eight-week intervention in MDD patients [17], which might be due to the longer intervention period. Hence, despite mixed results in literature [54] and the clear need of more research on the precise mechanisms of probiotics, our results dovetail with various studies showing beneficial effects of probiotics in depressive symptoms and underscore the potential of probiotics as add-on treatment in depression. Generally, the choice of the probiotics formulation and its dose is of great importance. As there is no clear evidence yet which bacteria specifically help to alleviate depressive symptoms, the choice of easily accessible probiotics is eligible. However, next generation probiotics with identified precise mechanisms of action might replace them in the future.

Next to the improvement of the depressive symptoms, the administered probiotics also modulated patients’ gut microbiota. Even though we could not confirm our hypothesis that probiotics would significantly decrease the prevalence of the *Bacteroides* 2 enterotype, we found that probiotics maintained diversity and richness and that enterotypes related to health were altered during the study intervention. However, further longitudinal analysis with denser time series should be done to confirm our hypothesis. These results indicate that probiotics might halt the decay of the bacterial community which occurs in depression during the invention period [8], but a longer follow-up of the subjects would be required to check the effectiveness in a longer period. The decrease of the healthy *Prevotella* enterotype [13] in the placebo but not the probiotics group further implies that probiotics help to maintain a healthy bacterial community. The probiotic administration increased the abundance of *Lactobacillus* strains which was the only significant time*group interaction. The antidepressant effect of the probiotics could be related to the abundance increase of these species, which is congruent with previous reports [55]. For instance, the *Lactobacillus* genus can produce GABA in mouse studies [56], and it has been shown to reduce stress-induced corticosterone and anxiety- and depression-related behavior [57]. Indeed, the increase of the genus was associated with reduced depressive symptoms as measured with the HAM-D and BDI. The potential of species of the *Lactobacillus* genus as add-on therapy has been demonstrated in different works by its capacity to enhance the integrity of the intestinal barrier, improve immune tolerance, reduce the bacteria translocation [58] and bring beneficial effects on anxiety and depression-related behaviors [36, 59]. Strains of the *Lactobacillus* genus are able to produce short-chain fatty acids (SCFA) such as acetate, butyrate, and propionate [60–63], which play an important role in maintaining host health and exert beneficial effects without inducing remodeling of the gut microbiome [64]. For instance, the ingestion of the probiotics *Lactobacillus casei* increased the levels of species of the *Lactobacillus* and *Bifidobacteria* genera and reduced anxiety symptoms in subjects with chronic fatigue syndrome [65]. Significant time*group interactions were only found for the *Lactobacillus* genus even though the two different ASVs Ruminococcaceae 264, Lachnospiraceae 318 in the placebo group showed significant increases in their abundance which were partly associated with depression scales. Complementing our results showing an increase in the placebo group, the ASV of the family *Ruminococcaceae* has recently shown to be reduced in subjects with mental disorders [12].

Our findings regarding effects on emotion processing were not in line with our a priori hypothesis and we could not replicate previous findings regarding amygdala responses in relation to symptoms’ improvement in IBS patients [21]. Nonetheless, our findings support the claim of a beneficial effect of probiotics in addition to TAU as the calcarine sulcus and the putamen are both affected in depression [66, 67]. Especially, the activation decrease in the putamen during neutral face processing indicates a probiotics’ beneficial effect on emotional information processing. Although the putamen’s role in depression is not very well established yet, its activation is shown to be aberrant in response to facial emotion processing [68] and strongly modulated by emotional valence [69]. Depressive patients show a hyperactivation while processing emotionally negative stimuli and a hypoactivation while processing emotionally positive stimuli compared to healthy controls [70]. Putamen’s hyperactivation evoked by emotionally negative stimuli is postulated to contribute to negativity biases often found in MDD patients [69, 71] such as perceiving emotionally neutral faces as rather emotionally negative faces [72]. Based on these findings, the reduced activation in the putamen found here can be interpreted as a shift in emotional valance of neutral faces. Neutral faces are perceived as more neutral after the intervention than before in the probiotics group; resulting in the observed activation decrease in the putamen. This interpretation is in line with previous findings that a three-week administration of prebiotics, fibers that promote growth of beneficial gut bacteria, reduces the attentional vigilance to negative compared to positive information in healthy women [73]. Thus, our findings implicate that probiotics modify the negativity bias in emotional face processing and meet the main requirement of a successful treatment in depression defined by altering negative affective biases [71, 74].

Our study has some limitations that need to be addressed in the future. Although we found strong evidence for the beneficial effect of probiotics in depression, our sample size is relatively small. While the intervention product was supplied by nursing personnel, compliance was not perfect, and cases with low compliance were excluded. Thus, future large-scale studies are needed to replicate and validate our findings. The inhomogeneous gut microbiota results might be caused by the variety of different probiotic strains used in studies and the complexity of their mode of action. In addition, it would be important to examine the interactions of probiotics with general antidepressant medication used in TAU to test if synergisms generally exist or if probiotics’ beneficial effects depend on specific antidepressants. It would further be interesting to examine whether changes in brain structure and function become more apparent after four weeks of the intervention as seen in the behavioral data. The increased behavioral effect after eight weeks implies that changes in the brain might also be greater after eight weeks; particularly for investigating changes in the brain structure, the four-week period is a very short time.

In conclusion, our results suggest that an add-on probiotic treatment improves depressive symptoms and maintains healthy enterotypes, species richness and increases specific health related bacterial taxa. On a neural level, probiotics alter negative biases and emotional valence additionally to TAU for depression. The present findings highlight the role of the microbiota-gut-brain axis in MDD and emphasizes the potential of microbiota-related treatment approaches as accessible, pragmatic, and non-stigmatizing therapies to improve the effectiveness of current treatments in depression.

## Supplementary information

Supplemental Material

## Data Availability

The datasets generated and analyzed during the current study are available from the corresponding author on reasonable request.
