# Supplementary material for: Clinical, gut microbial and neural effects of a probiotic add-on therapy in depressed patients: a randomized controlled trial
Source: Transl Psychiatry. 2022 Jun 3;12:227. doi: 10.1038/s41398-022-01977-z (PMC9163095; doi:10.1038/s41398-022-01977-z)
Supplement: Supplementary file 1 — Supplemental Material [file 41398_2022_1977_MOESM1_ESM.doc]

**Clinical, gut microbial and neural effects of a probiotic add-on therapy in depressed patients: A randomized-controlled trial**

**Supplementary information (SI)**

**Supplementary Methods**

**Supplementary Figure 1**. Study design. MRI = magnetic resonance imaging; HAM-D = Hamilton Rating Scale for Depression; BDI = Beck Depression Inventory; GSRS = Gastrointestinal Symptom Rating Scale; STAI1 = State-Trait Anxiety Inventory 1.

Medication

To monitor treatment as usual, medication intake was extracted for each patient from the hospital information system. Antidepressants and antipsychotics were transformed into dose equivalents based on the defined daily dose (DDD) method, which is described as the “*assumed average maintenance dose per day for a drug used for its main indication in adults»*.^1^ For each patient, detailed information is depicted in Table S1. Different antidepressants and antipsychotics were summarized using the DDD. Furthermore, the specific period of intake was considered as the drugs were not necessarily administered over the whole study period.

**Supplementary Table 1.** Antidepressant and antipsychotic medication per patient in mean defined daily dose (DDD) over the four-week intervention period. Only patients that finished the intervention are included.

| Group | Patient ID | Name | DDD |
| --- | --- | --- | --- |
| Probiotics | Patient 1 | mirtazapine, fluoxetine, trimipramine, lithium, olanzapine, chlorprothixene, quetiapine | 3.45 |
|  | Patient 2 | venlafaxine, lithium, quetiapine | 0.76 |
|  | Patient 3 | venlafaxine, quetiapine | 2.33 |
|  | Patient 4 | lithium, quetiapine | 0.75 |
|  | Patient 5 | bupropion, trazodone | 1.47 |
|  | Patient 6 | venlafaxine, trazodone, quetiapine | 2.47 |
|  | Patient 7 | quetiapine | 0.01 |
|  | Patient 8 | sertraline, quetiapine | 3.22 |
|  | Patient 9 | escitalopram | 2.00 |
|  | Patient 10 | venlafaxine, quetiapine | 4.02 |
|  | Patient 11 | duloxetine, quetiapine | 1.12 |
|  | Patient 12 | lithium, citalopram, quetiapine, olanzapine | 4.33 |
|  | Patient 13 | duloxetine, quetiapine | 1.04 |
|  | Patient 14 | duloxetine | 0.50 |
|  | Patient 15 | mirtazapine, sertraline, quetiapine | 5.26 |
|  | Patient 16 | venlafaxine, quetiapine | 3.10 |
|  | Patient 17 | sertraline, mirtazapine | 3.25 |
|  | Patient 18 | bupropion, trimipramine, clotiapine, pipamperone | 0.93 |
|  | Patient 19 | duloxetine | 0.50 |
|  | Patient 20 | bupropion, quetiapine | 1.13 |
|  | Patient 21 | duloxetine | 1.00 |
| Placebo | Patient 1 | vortioxetine, quetiapine | 1.57 |
|  | Patient 2 | sertraline, vortioxetine, clotiapine | 1.91 |
|  | Patient 3 | duloxetine, olanzapine | 3.00 |
|  | Patient 4 | venlafaxine, olanzapine, clotiapine | 3.02 |
|  | Patient 5 | duloxetine, risperidone, quetiapine | 1.03 |
|  | Patient 6 | agomelatine | 1.74 |
|  | Patient 7 | venlafaxine | 2.73 |
|  | Patient 8 | mirtazapine, quetiapine | 1.06 |
|  | Patient 9 | bupropion | 1.00 |
|  | Patient 10 | bupropion | 0.45 |
|  | Patient 11 | vortioxetine | 1.00 |
|  | Patient 12 | fluoxetine, olanzapine | 1.45 |
|  | Patient 13 | escitalopram, olanzapine | 2.97 |
|  | Patient 14 | lithium, olanzapine, pipamperone | 0.77 |
|  | Patient 15 | venlafaxine, vortioxetine, olanzapine | 3.99 |
|  | Patient 16 | duloxetine | 1.55 |
|  | Patient 17 | mirtazapine | 1.50 |
|  | Patient 18 | trazodone, bupropion | 0.60 |
|  | Patient 19 | trazodone, bupropion, duloxetine | 1.30 |
|  | Patient 20 | escitalopram | 2.00 |
|  | Patient 21 | escitalopram, lithium | 4.00 |
|  | Patient 22 | venlafaxine, mirtazapine, bupropion, clotiapine | 2.61 |
|  | Patient 23 | vortioxetine, quetiapine, olanzapine, chlorprothixene | 1.54 |
|  | Patient 24 | fluoxetine, agomelatine, pipamperone, quetiapine | 5.26 |
|  | Patient 25 | vortioxetine | 2.07 |
|  | Patient 26 | vortioxetine, clotiapine, quetiapine | 2.27 |

*Notes*. DDD = defined daily dose.

Study procedure

Patients had the right to withdraw from the study without being obliged to give reason. Adverse events challenging the health of the patients, severe protocol violations or administrative troubles resulted in withdrawal in the best interest of the patient. Block randomization was performed in a 1:1 ratio by an independent researcher using a computer-based randomization algorithm to avoid systematic biases. Therefore, investigators and assessors were blinded during data collection and analysis. Patients were informed about the allocation after the follow-up assessment by an independent researcher.

Statistical analysis of clinical measures

In a first step, analyses were run on an intention-to-treat (ITT) basis with all participants that completed the post-intervention assessment. In a modified ITT analysis (mITT),^2^ only patients with compliance >65% were included. This cut-off was based on evidence showing that patients receiving antidepressant medication took in average 65% of the prescribed amount.^3^ Single missing values in questionnaires were imputed with the k-nearest-neighbor method.^4^ HAM-D and BDI total scores over all time points were sqrt-transformed since distributions were non-normal. GSRS scores were log-transformed due to a strong negative skew. All statistical analyses on behavioral data were performed using R Version 3.6.3 including packages such as ‘lme4’.^5^ Treatment response was dichotomized according to the Clinical Global Impression (CGI) criteria as a change >57% in HAM-D scores compared to baseline measures.^6^

Gut microbiome

*Bacterial DNA extraction and sequencing*

The fecal DNA was extracted following the protocol described in Falony et al..^7^ Summarily, DNA was extracted from 150-200mg of the frozen samples using MagAttract PowerMicrobiome DNA/RNA KF kit (QIAGEN, Hilden, Germany) following the manufacturer's instructions. The V4 region of 16S rRNA genes was amplified using the 515F /806R primer pair and purified using the QIAquick PCR Purification Kit. Sequencing was performed using the Illumina MiSeq platform (MiSeq Reagent Kit v2, Illumina, San Diego, USA).

*16s rRNA data processing*

Amplicon data from the 16S rRNA gene was analyzed following the DADA2 pipeline specifications.^8^ Briefly, the first 30bp were removed, and the sequence length was set to 130bp and 200bp for the forward and reverse strands, respectively. The sequence error rate, dereplications, the inferred composition of the sample, and the chimera removal were done using the DADA2 default parameters. The taxonomic assignation was done using the DADA2 RDP implementation (R packages “dada2” function “assignTaxonomy”) with the rdp_train_set_16 as reference, similarly the amplicon sequence variant (ASV) annotation was done using the [GTDB_bac120_arc122_ssu_r202_Species](https://zenodo.org/record/4735821/files/GTDB_bac120_arc122_ssu_r202_Species.fa.gz?download=1) trainset (R packages “dada2” function “addSpecies”). The relative abundance was presented at the ASV level and summarized to the genus level.

*Microbial load measurement*

The microbial load of the study cohort was measured by flow cytometry as described previously.^9^ Briefly, 200-250 mg frozen (-80°C) fecal aliquots were diluted in saline solution (0.85% NaCl; VWR International, Germany) and filtered using a sterile syringe filter (pore size of 5 µm; Sartorius Stedim Biotech GmbH, Göttingen, Germany). Next, 1 mL of the microbial cell suspension obtained was stained with 1 µL SYBR Green I (1:100 dilution in DMSO; Thermo Fisher Scientific, Massachusetts, USA) and incubated for 15 min in the dark at 37°C. The flow cytometry analysis was performed using a C6 Accuri flow cytometer (BD Biosciences, New Jersey, USA) based on Prest et al..^10^ Fluorescence events were monitored using the FL1 533/30 nm and FL3 >670 nm optical detectors. The BD Accuri CFlow software was used to gate and separate the microbial fluorescence events on the FL1/FL3 density plot from the fecal sample background. A threshold value of 2000 was applied on the FL1 channel. Based on the exact weight of the aliquots analyzed, cell counts were converted to microbial loads per gram of fecal material.

*Quantitative microbiome profiling*

The quantitative microbiome profiling (QMP) matrix was built as described by Vandeputte and colleagues.^9^ In brief, samples were downsized to even sampling depth, defined as the ratio between sampling size (16S rRNA gene copy number-corrected sequencing depth) and microbial load (the average total cell count per gram of frozen fecal material). 16S rRNA gene copy numbers were retrieved from the rRNA operon copy number database rrnDB33. The final matrices were represented as the "QMP" matrix and the "even sample depth rarefied matrix", which is the number of reads per sample rarefied according to the sampling depths determined by the sample's cell counts.

*Fecal moisture content*

The fecal moister content was determined as the percentage of mass loss after lyophilization from 0.2g frozen aliquots of non-homogenized fecal material as previously done.^7^

*Fecal calprotectin measurement*

Fecal calprotectin concentrations were determined using the fCAL ELISA Kit (Bühlmann, Amherst, USA). The measurements were done on frozen fecal material (-80°C).

*Enterotyping*

The 16s rRNA bacterial profiles were collapsed at the genus level and integrated along with the FGFP cohort as done in the previous work.^7^ The identification of the enterotypes was accomplished with the Dirichlet-multinomial Model (DMM) approach in R (library "DirichletMultinomial" function "dmn"). To compare enterotype distributions between depressed patients and healthy subjects, a group of 93 healthy subjects that was matched by age, BMI and sex with the study sample (sex χ2(1)=0, p=1; BMI W=5225, p=.96; age W=5168.5, p=0.94) was taken from the Belgian Flemish Gut Flora (FGFP) cohort.^11^

*Diversity analysis*

Diversity analysis was performed using the R statistical software (v3.6.3). The beta diversity analysis from the 16S rDNA amplicon sequence variant (ASV) data was estimated. The Bray-Curtis index (library "Vegan", function "vegdist") was used to estimate the dissimilarities between samples in the QMP even sampling depth ASV table. Low frequent ASV data (80% of zero data) were removed before the dissimilarity estimation. A distance-based redundancy analysis (dbRDA) (library "Vegan" function "capscale") was performed to reduce dimensionality in the taxonomic and functional distance matrix. The Permutational Multivariate Analysis of Variance Using Distance Matrices (ADONIS test) (library "vegan" function "adonis") clinical and metadata variables. Clinical measures were correlated into the ordination using the function “envfit” (library "vegan"). The adonis and envfit p-values were adjusted using the Benjamini-Hochberg method (library "stats" function "p.adjust").

Observed richness, Shannon and Inverse Simpson index (library "phyloseq" function "estimate_richness") and Pielou's evenness (library "microbiome" function "evenness") indices were estimated at the genus level for each sample.

*Statistical analysis of gut microbiota data*

Differences in the frequency of enterotypes was determined through Chi-squared tests (library "stats" function "chisq.test") and the difference in the frequency of the enterotypes over time within the placebo and probiotic group by symmetry tests for paired contingency tables (library "rcompanion" function "nominalSymmetryTest"); p-values were adjusted using Benjamini-Hochberg's correction (library "stats" function "p.adjust"). The effect of the intervention on enterotypes was examined using a binomial generalized linear model (library "lme4" function "lmer"). The dependent variable was the enterotype two-level categorization (enterotype and non-enterotype) for all four different enterotypes. The fixed effect was the time*group interaction, moisture, calprotectin, sex, body-mass index (BMI), and age; and the subject ID was set as random intercept. An ANOVA test and the AIC determined the significance and feature selection model (library "car" function "Anova"). The effect of probiotics in reducing Bacteroides 2 prevalence was estimated by means of an odds ratio (OR) between the frequency of the Bacteroides 2 enterotype of the placebo and probiotic group and the FGFP matched controls (library "epitools" function "oddsratio").

Associations between bacterial taxa (ASV and genus) and time were estimated using a zero-inflated mixed effect negative binomial model if the taxa prevalence were between 20 and 80%, and mixed effect negative binomial model, if the taxa prevalence were above 80%, as suggested by Zhang and Yi.^12^ The model considers time as fixed effect; the subject ID was modeled using a random intercept (library "NBZIMM" function "mms" and library "glmmTMB" function "glmmTMB"). The zero-inflation parameter was determined using a single zero-inflation parameter applied to all observations (~1). An ANOVA test (library "car" function "Anova") determined the significance of the overall effect of time over the taxa; meanwhile, the Wald-test was used to determine the significance of the three levels of the time variable (library "base" function "summary"). All p-values were adjusted using Benjamini-Hochberg's correction (library "stats" function "p.adjust"), a taxon was considered to be associated with time if the ANOVA and all the Wald-test corrected p-values were significant and if the mean abundance of the post-intervention and follow-up were congruently higher or lower in comparison to the baseline. The taxa*time interactions were confounded by fecal moisture, sex, BMI, and age using the step function (library "stats" function "step"). The analyses were done independently for the placebo and probiotic group. The taxonomic time*group comparison was modelled using the before mentioned strategy but setting the time*group interaction as the fixed effect and the subject ID as random intercept (library "glmmTMB" function "glmmTMB").

A similar approach was made to estimate the associations between bacterial taxa and behavioral measures and fecal calprotectin. Taxa were modelled as a mixed effect negative binomial or as a mixed effect zero-inflated negative binomial model depending on its zero abundance as described above. The model considers clinical measures and fecal calprotectin as fixed effects; the subject ID as random effects (library "glmmTMB" function "glmmTMB"). Independent models were done for the HAM-D, GSRS, BDI, STAI1 and fecal calprotectin. An ANOVA test (library "car" function "Anova") determined the significance of taxa interactions. All p-values were adjusted using Benjamini-Hochberg's correction (library "stats" function "p.adjust"). The taxon-variable associations were confounded by fecal moisture, sex, BMI, and age using the step function (library "stats" function "step"). Analyses were done separately per group. All statistical analyses were done using R (v3.6.3).

*Visualization of gut microbiota analyses*

The taxonomic summarization at the Phylum level was visualized into a barplot. All visualizations such as these barplots, boxplots and PcoA were done using the ggplot package in R.

Brain structure and function

*Participants for imaging*

For the analyses of the imaging data, data of 32 patients were available after excluding non-compliant patients (N_probiotics_=14, N_placebo_=18). Furthermore, an additional sample of 20 healthy controls (age: 40.25 ±10.91 years; 4 women) was used to be able to examine which structures are generally affected in the depressive patients compared to healthy people.

*Image acquisition and data analysis*

Anatomical and functional image acquisition was carried out on a 3T Siemens Magnetom Prisma whole-body scanner (Siemens Medical Solutions, Erlangen, Germany) with a 20-channel head coil. For structural data, an anatomical T1-weighted image acquisition followed a three-dimension (3D) magnetization-prepared rapid gradient-echo (MP-RAGE) sequence pulse^13^ with a spatial resolution of 1×1×1 mm^3^ (slice thickness: 1.0 mm, 176 sagittal slices; time of repetition (TR) = 2000ms; echo time (TE)=3.37ms; flip angle (FA)=8°; field of view (FOV)=256×256mm^2^). To reveal structural brain changes due to the probiotic intervention, a voxel based morphometry was performed using the Computational Anatomy Toolbox (CAT) toolbox.^14^ A flexible factorial design with the factors subject ID, group and time was used for data analysis.

For functional data, functional T2*-weighted images were acquired using a blood-oxygen-level-dependent (BOLD) sensitive, interleaved diffusion-weighted echo planar imaging (EPI) sequence with a spatial resolution of 3×3×3mm^3^ (slice thickness: 3.0mm, 39 transversal slices; TR=2500ms; TE=30ms; FA=82°; FOV=228×228mm^2^). The task included 10 different facial identities, each presented twice in the categories neutral, 50% and 100% intensity of fear; resulting in 60 faces in total. Faces were presented for 2 sec in a pseudo-random order avoiding successive presentations of the same face. Between the faces, a fixation cross was presented for a duration of 2-8 sec. As task, patients had to indicate the face’s gender.

Data analysis was performed with SPM12 (http://www.fil.ion.ucl.ac.uk/spm/). All volumes were realigned to the first volume, coregistered to the anatomical volume, normalized to the MNI305 T1 template and finally smoothed with a 6 mm (FWHM) isotropic Gaussian kernel. During model specification, onset times for each trial of neutral, 50%, and 100% fearful faces (event-related design) and the duration of the fixation cross (block design) were convolved with a canonical hemodynamic response (HPR) function. Serial correlations were removed with a first-order autoregressive model, and a high-pass filter (128 sec) was applied to remove low-frequency noise. The six movement parameters were further included as nuisance covariates.

Each trial of the neutral, 50% and 100% fearful faces was subsequently contrasted against the fixation cross.^15^ Afterwards, the activation changes over time were calculated for each face category by subtracting the activation contrasts in the post-intervention from the baseline. These subject-specific contrast images were propagated to the second-level analysis where a full factorial design with the two factors group (probiotic vs placebo) and faces (neutral, 50% and 100% fearful) was used. Moreover, t-tests for neutral, semi-fearful and fearful faces were conducted to reveal activation changes between MDD patients and healthy controls during fearful face processing.

For imaging analyses regarding probiotics and placebo groups, we set the intensity threshold of the peak-voxel to a p-value of 0.001, uncorrected, and the minimal cluster size threshold k to 10 voxels. Additionally, we demanded a p-value of 0.05, familywise error correct, on cluster level to identify a cluster as significant. For the contrasts between patients and healthy controls we set the intensity threshold of the peak-voxel to a p-value of 0.05, familywise error corrected, and the minimal cluster size threshold k to 10 voxels.

**Supplementary results**

Missing data analysis

The 12 patients who dropped out of the study during the intervention had equal HAM-D baseline scores than patients who completed the trial (*t*(17.38)=-0.64, *p*=.53). Furthermore, dropouts were not associated with gender (*χ2*(1, 59)=0.06, *p*=.80) and age (*t*(17.38)=1.66, *p*=.12).

**
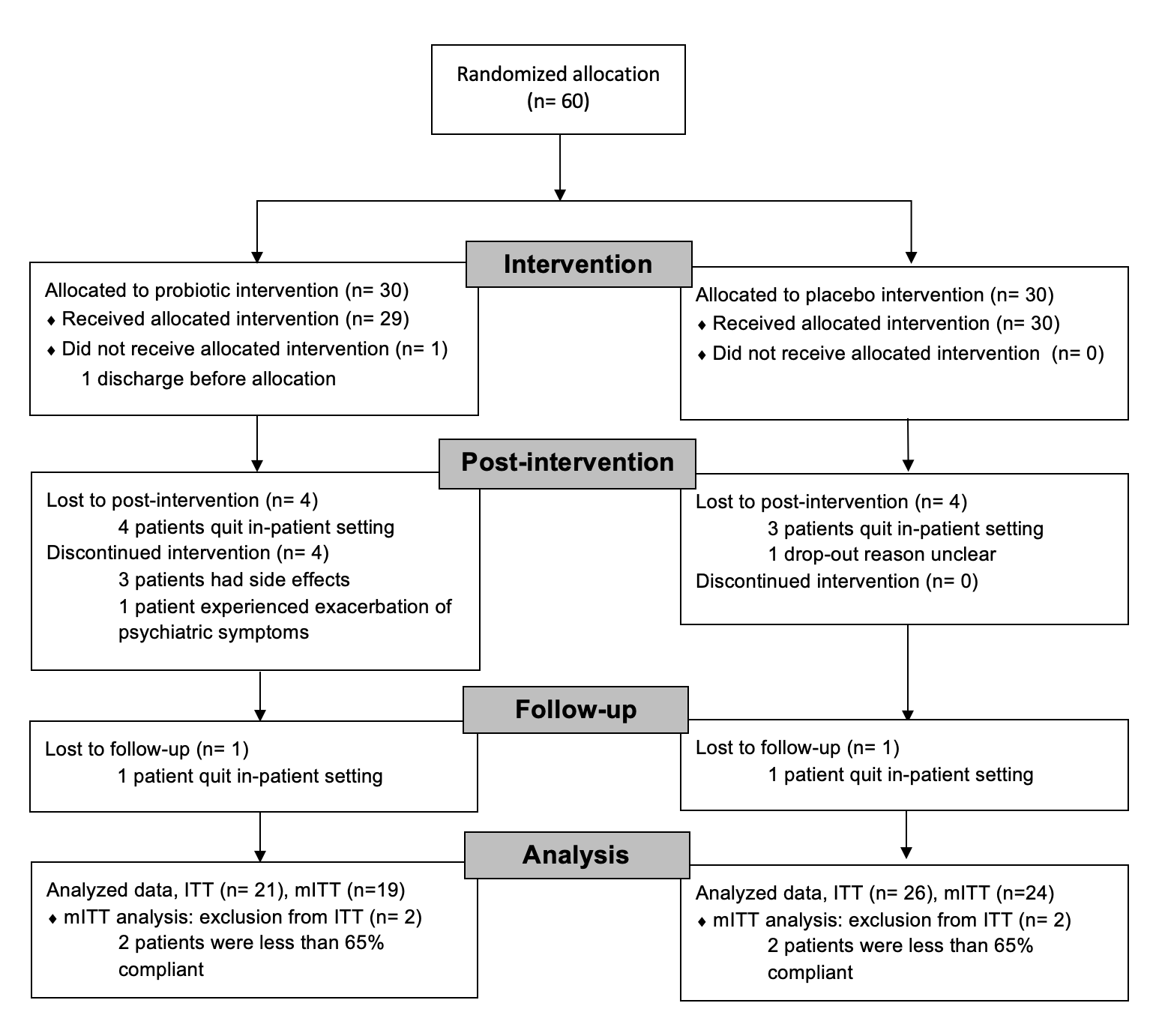
**

**Supplementary Figure 2.** CONSORT diagram of participants of the probiotics study. ITT = intention-to-treat; mITT = modified intention-to-treat.

**
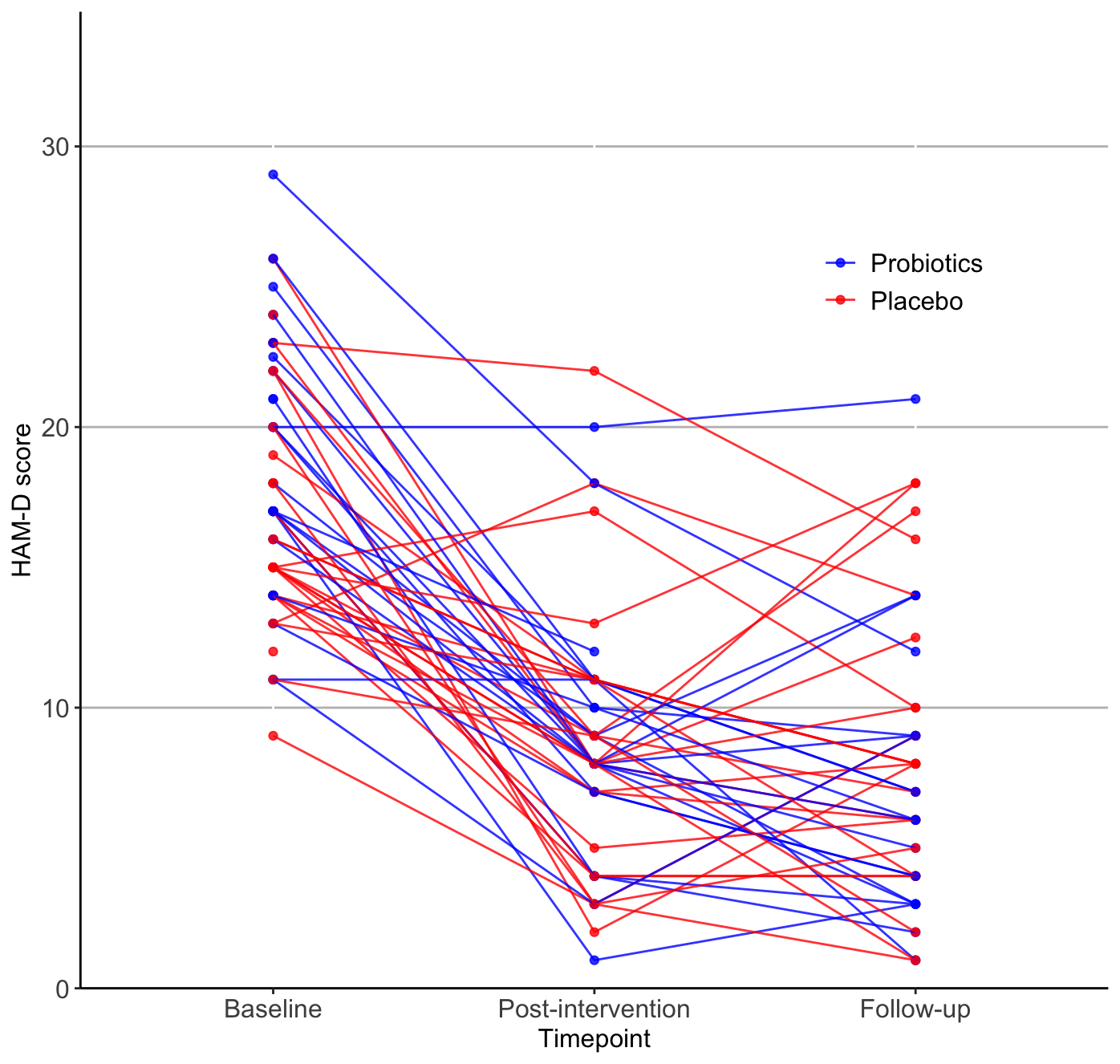
**

**Supplementary Figure 3.** Individual trajectories of depressive symptoms (HAM-D) in the intention-to-treat (ITT) sample. HAM-D = Hamilton Rating Scale for Depression.

**Supplementary Table 2.** Results of ANOVA over linear mixed models predicting depression scores (HAM-D) for ITT and mITT samples.

|  | HAM-D^a^ | | | | | | | |
| --- | --- | --- | --- | --- | --- | --- | --- | --- |
|  | ITT sample | | | | mITT sample | | | |
| Main effect | df | MS | F | *p* | df | MS | F | *p* |
| Group | 1 | 0.00 | 0.001 | .98 | 1 | 0.08 | 0.23 | .64 |
| Time | 2 | 34.65 | 98.28 | **<.001** | 2 | 34.27 | 100.56 | **<.001** |
| Time*group | 2 | 0.79 | 2.23 | .11 | 2 | 1.16 | 3.4 | **.04** |

*Notes.* ^a^ sqrt- transformed; HAM-D = Hamilton Rating Scale for Depression; ITT = intention-to-treat; mITT = modified intention-to-treat; *df* = degrees of freedom; MS = mean squares.

**Supplementary Table 3.** Results of ANOVA over linear mixed models predicting self-reported depressive symptoms (BDI) for ITT and mITT samples.

|  | BDI^a^ | | | | | | | |
| --- | --- | --- | --- | --- | --- | --- | --- | --- |
|  | ITT sample | | | | mITT sample | | | |
| Main effect | *df* | MS | *F* | *p* | *df* | MS | *F* | *p* |
| Group | 1 | 0.071 | 0.12 | .73 | 1 | 0.38 | 0.62 | .43 |
| Time | 2 | 32.99 | 54.68 | **<.001** | 2 | 31.46 | 51.46 | **<.001** |
| Time*group | 2 | 0.267 | 0.44 | .64 | 2 | 0.73 | 1.2 | .31 |

*Notes.* ^a^ sqrt- transformed; BDI = Beck Depression Inventory; ITT = intention-to-treat; mITT = modified intention-to-treat; *df* = degrees of freedom; MS = mean squares.

**Supplementary Table 4.** Results of ANOVA over linear mixed models predicting self-reported anxiety (STAI1) for ITT and mITT samples.

|  | STAI1 | | | | | | | |
| --- | --- | --- | --- | --- | --- | --- | --- | --- |
|  | ITT sample | | | | mITT sample | | | |
| Main effect | *df* | MS | *F* | *p* | *df* | MS | *F* | *p* |
| Group | 1 | 4.19 | 0.05 | .82 | 1 | 33.28 | 0.402 | .53 |
| Time | 2 | 1005.9 | 12.56 | **<.001** | 2 | 824.79 | 9.983 | **<.001** |
| Time*group | 2 | 75.0 | 0.94 | .40 | 2 | 38.25 | 0.463 | .63 |

*Notes.* STAI1 = State-Trait Anxiety Inventory 1; ITT = intention-to-treat; mITT = modified intention-to-treat; *df* = degrees of freedom; MS = mean squares.

**Supplementary Table 5.** Results of ANOVA over linear mixed models predicting gastrointestinal symptoms (GSRS) for ITT and mITT samples.

|  | GSRS^a^ | | | | | | | |
| --- | --- | --- | --- | --- | --- | --- | --- | --- |
|  | ITT sample | | | | mITT sample | | | |
| Main effect | *df* | MS | *F* | *p* | *df* | MS | *F* | *p* |
| Group | 1 | 0.13 | 0.34 | .56 | 1 | 0.03 | 0.51 | .48 |
| Time | 2 | 1.27 | 3.35 | **<.05** | 2 | 0.17 | 3.16 | **<.05** |
| Time*group | 2 | 0.42 | 1.11 | .33 | 2 | 0.07 | 1.3 | .28 |

*Notes.* ^a^log-transformed; GSRS = Gastrointestinal Symptom Rating Scale; ITT = intention-to-treat; mITT = modified intention-to-treat; *df* = degrees of freedom; MS = mean squares.


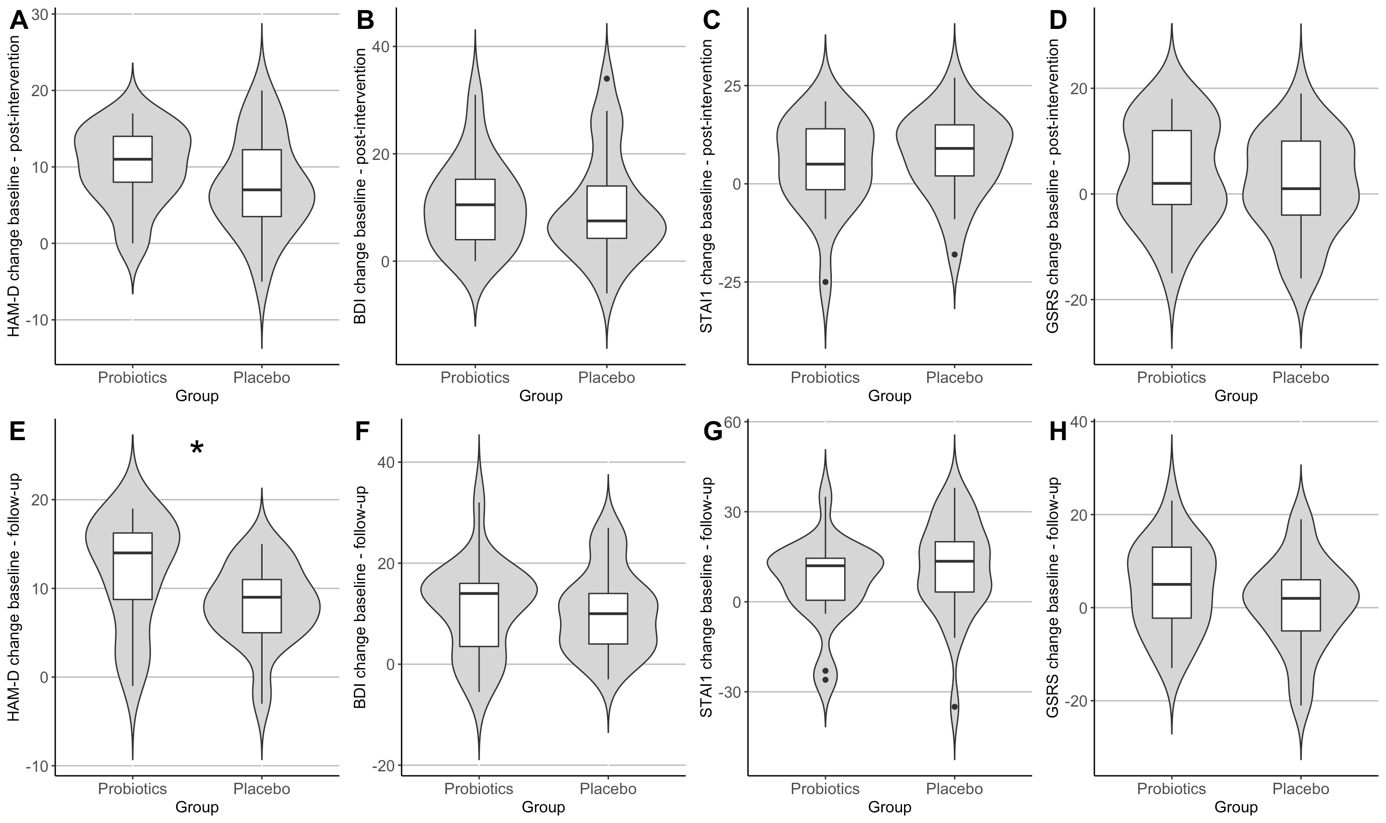


**Supplementary Figure 4.** Change scores from baseline to post-intervention (A, B, C, D) and from baseline to follow-up (E, F, G, H) of primary and secondary clinical outcomes in the intention-to-treat (ITT) sample. HAM-D = Hamilton Rating Scale for Depression; BDI = Beck Depression Inventory; STAI1 = State-Trait Anxiety Inventory 1; GSRS = Gastrointestinal Symptom Rating Scale.


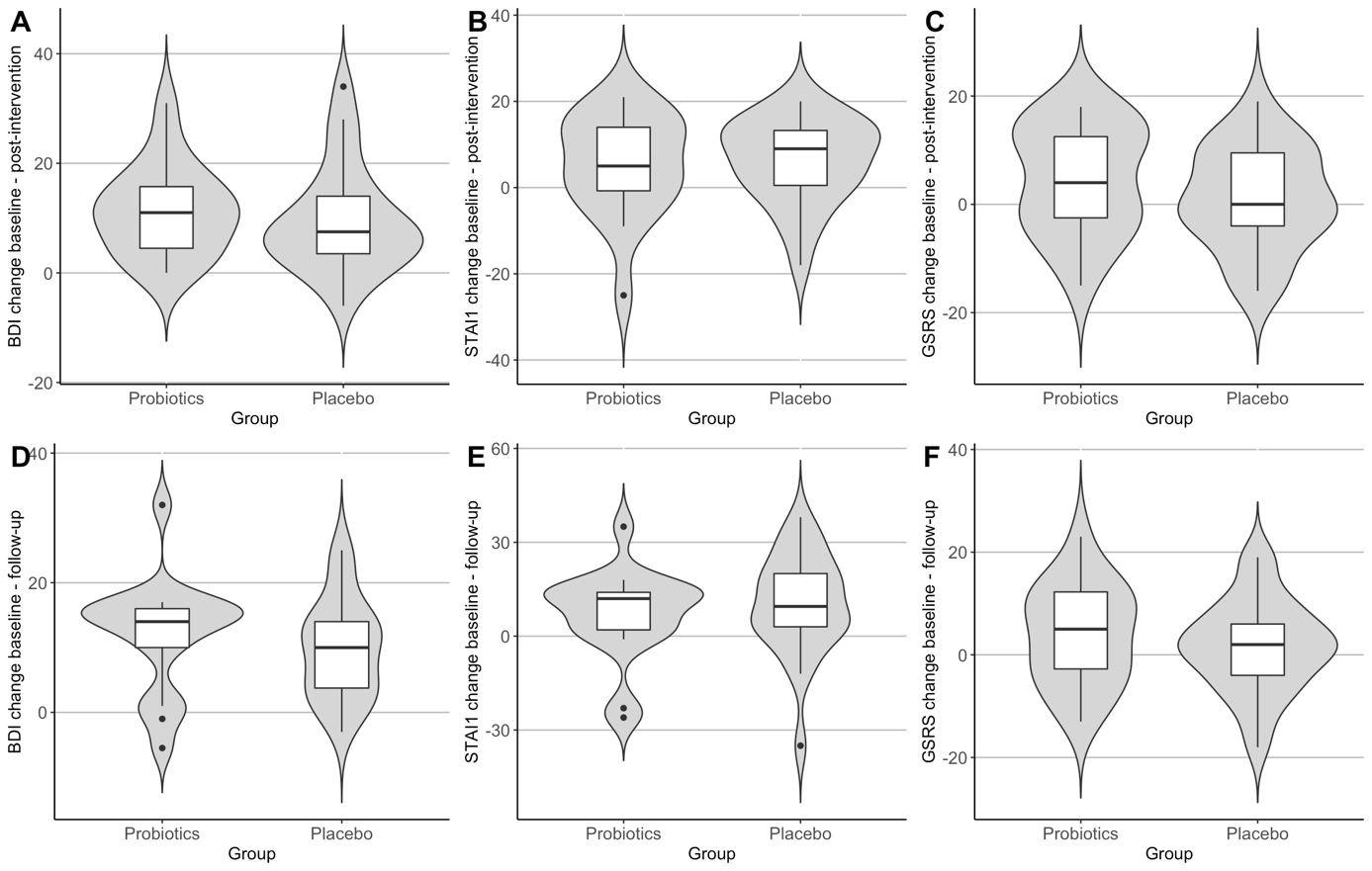


**Supplementary Figure 5.** Change scores from baseline to post-intervention (A, B, C) and from baseline to follow-up (D, E, F) of secondary clinical outcomes in the modified intention-to-treat (mITT) sample. BDI = Beck Depression Inventory; STAI1 = State-Trait Anxiety Inventory 1; GSRS = Gastrointestinal Symptom Rating Scale.

**Supplementary Figure 6.** Moisture, calprotectin and cell counts in fecal samples in both study groups and all time points.

**Supplementary Table 6.** Group comparisons of moisture, cell counts or calprotectin between both study groups at all time-points.

|  | Time point | Probiotics group | Placebo group | p | p-BH |
| --- | --- | --- | --- | --- | --- |
| Cells per gram of faeces,  mean (SD) | Baseline | 129849628868.111 (72011859905.177) | 121182026443.551 (60252192580.798) | 0.967 | 0.976 |
|  | Post-intervention | 133487145556.333 (88477574418.402) | 115298882513.253 (55271865891.772) | 0.685 | 0.976 |
|  | Follow-up | 138348498920.173 (81311664488.295) | 134999480238.176 (64100242202.505) | 0.976 | 0.976 |
| Fecal calprotectin, μg/g, mean (SD) | Baseline | 42.901 (81.021) | 47.267 (75.391) | 0.607 | 0.976 |
|  | Post-intervention | 30.819 (24.032) | 41.525 (70.191) | 0.311 | 0.976 |
|  | Follow-Up | 28.055 (18.453) | 43.505/ (75.033) | 0.717 | 0.976 |
| Moisture, %, mean (SD) | Baseline | 72.619 (6.396) | 72.978 (8.483) | 0.857 | 0.976 |
|  | Post-intervention | 74.521 (8.115) | 76.568 (6.796) | 0.512 | 0.976 |
|  | Follow-up | 67.505 (9.527) | 74.322(10.495) | 0.169 | 0.976 |

*Notes.* BH = Benjamini-Hochberg correction.


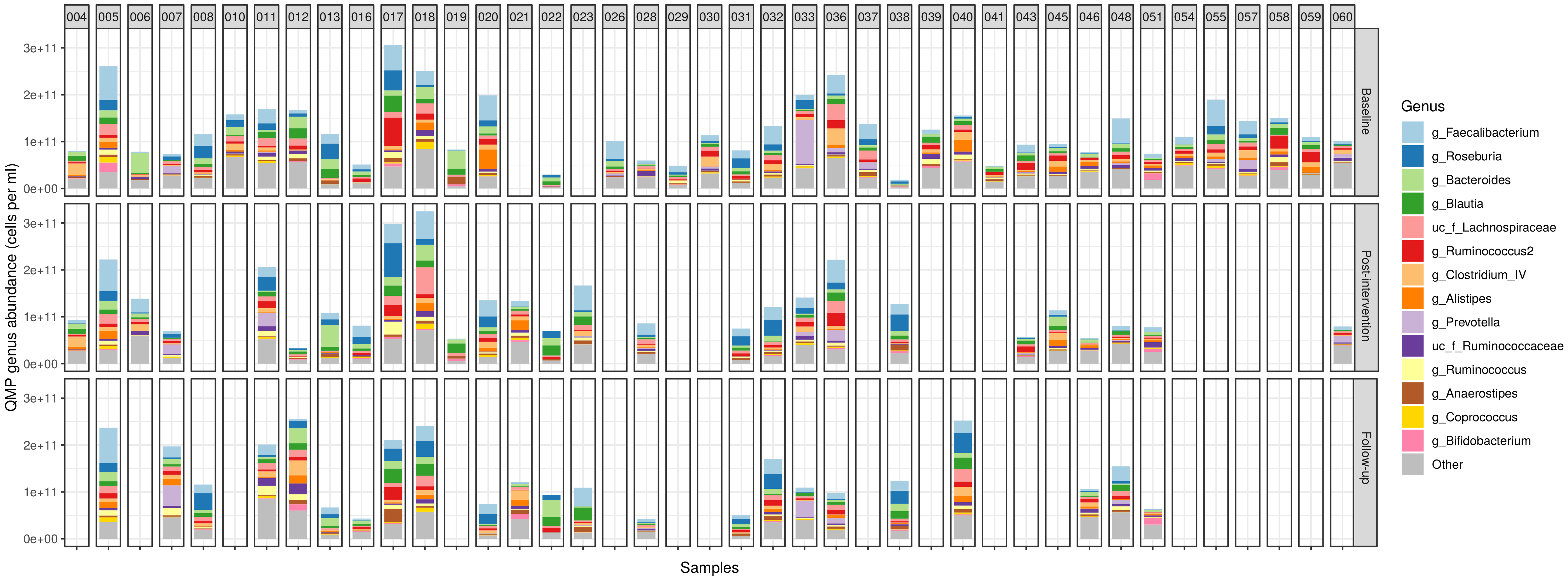


**Supplementary Figure 7.** Microbiome composition per subject and time point.

Enterotype distribution compared to healthy samples

The abundance of the Bacteroides 2 enterotype was higher in our study sample than in the FGFP sample (Supplementary Figure 8A) but when comparing the depressed patients with a matched subset of the FGFP, Bacteroides 2 prevalence was not significantly higher (χ^2^, adj p>.1) (Supplementary Figure 8B).

The OR between the study groups and the FGFP showed a non-significant decrease of the Bacteroides 2 enterotype in the probiotic group and contrary, the placebo showed a peak in the increase at the post-intervention (Supplementary Figure 9). However, the OR changed not significantly over time (OR χ^2^ adjusted p>0.1).


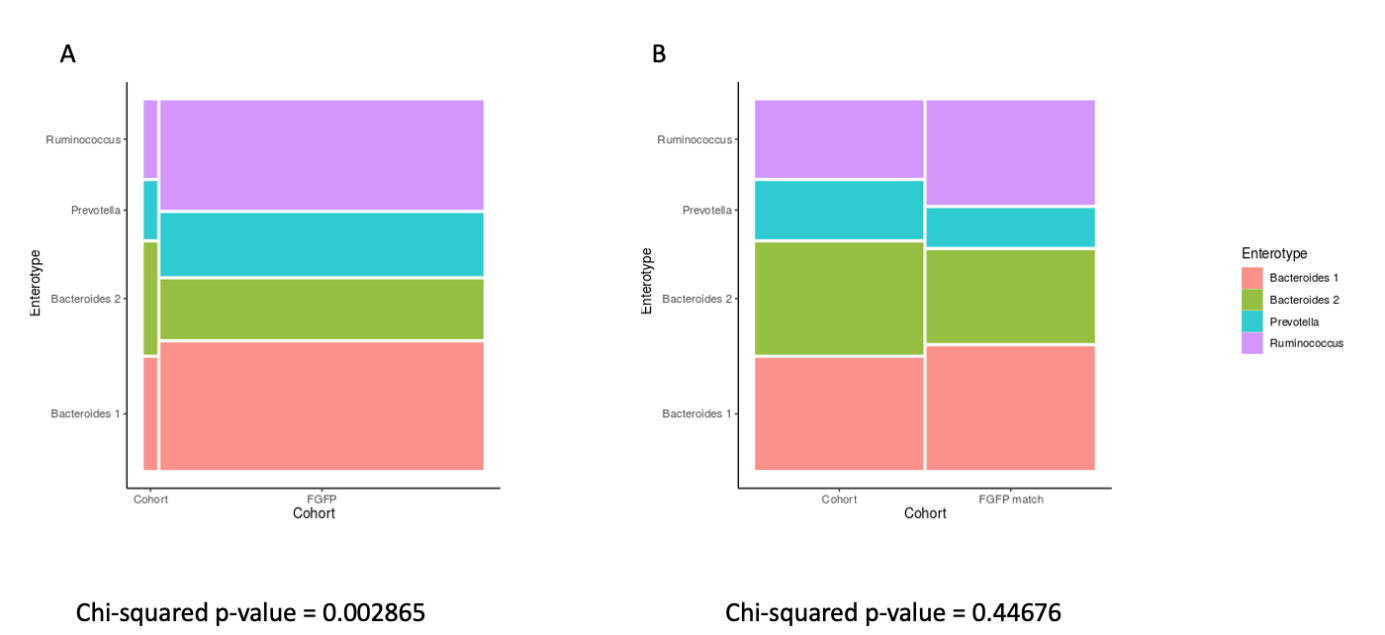


**Supplementary Figure 8**. Enterotype distribution of depressed subjects (combined probiotics and placebo group) compared to the healthy FGFP cohort (A) and the matched healthy sample (B).


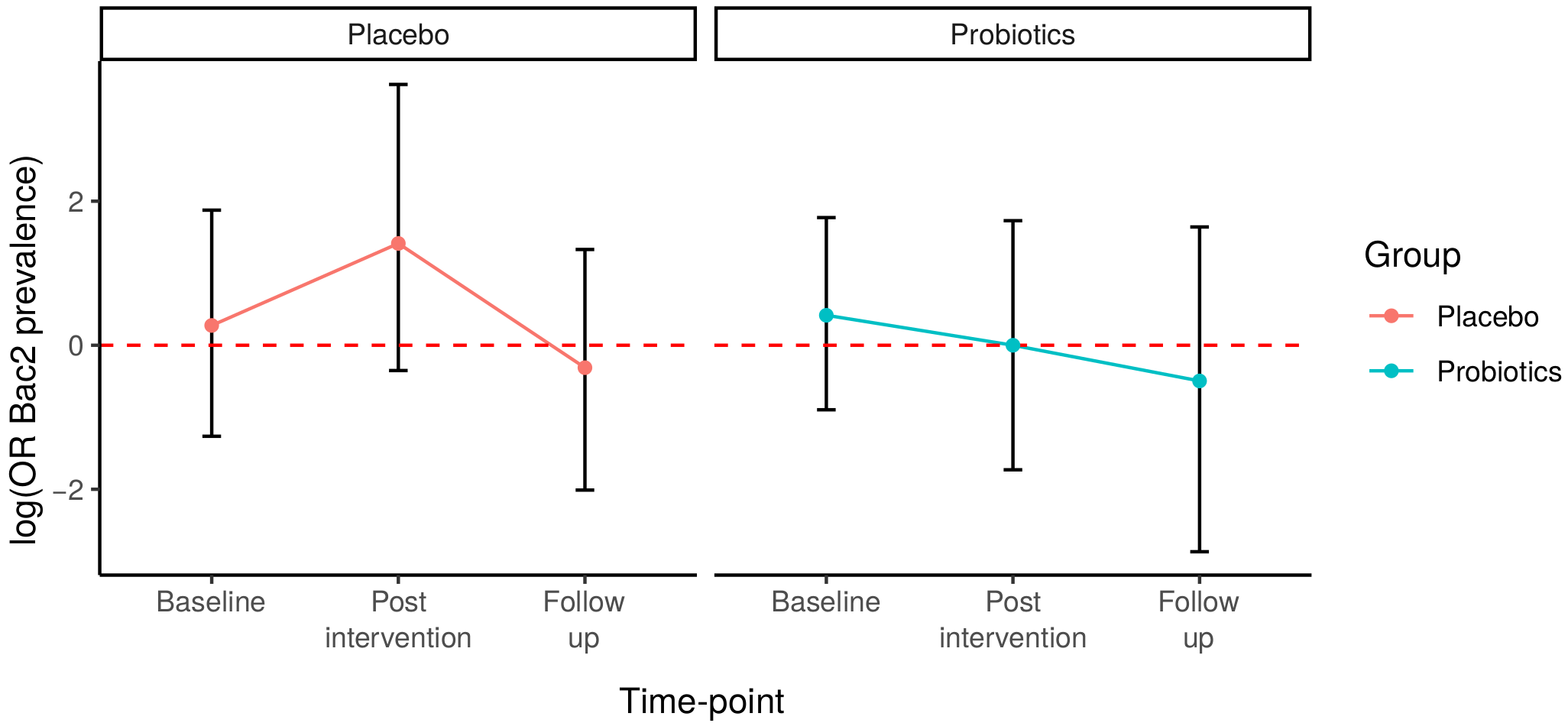


**Supplementary Figure 9.** Odds Ratio (OR) changes over time per study group. Y-axis is log-transformed.


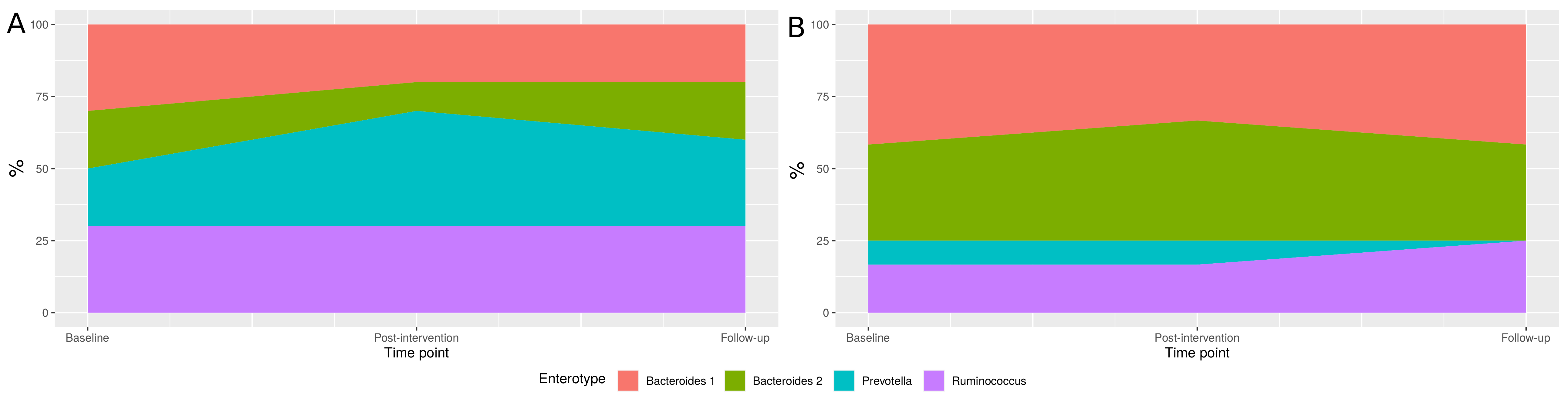


**Supplementary Figure 10.** Enterotype distribution over time in (A) the probiotics and (B) placebo group.

**Supplementary Figure 11.** Alpha-diversity indices such as (A) observed species, (B) inversed Simpson, (C) Pielou’s evenness and (D) Shannon over time in the two study groups.


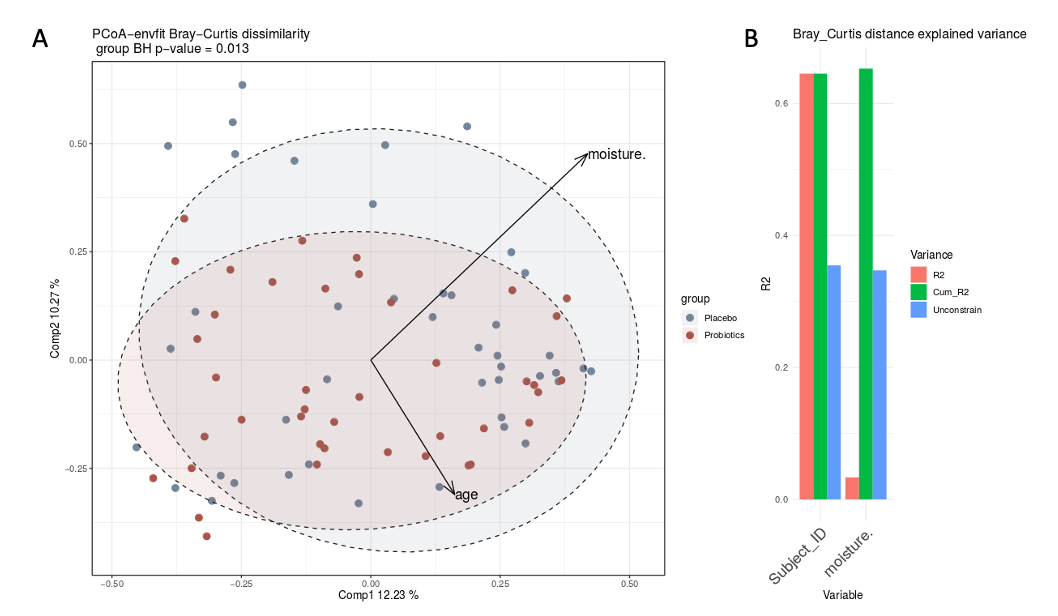


**Supplementary Figure 12.** Difference in the microbiome composition (beta-diversity) between the placebo and probiotics group. (A) Group differences and (B) explained variance of moisture and subject. BH = Benjamini-Hochberg correction; R^2^ = explained variance.

**Supplementary Figure 13.** Identified altered taxa in both study groups for all time points. The significance of the placebo and probiotic comparisons were carried out using Wilcoxon signed-rank test and the p-value adjusted using Benjamini-Hochberg method.

**Supplementary Table 7.** Explained variance of beta-diversity.

| Variable | F-model | R^2^ | p | p-BH |
| --- | --- | --- | --- | --- |
| Subject ID | 4.556 | 0.809 | 0.001 | 0.006 |
| Time*group | 0.806 | 0.049 | 0.947 | 1.000 |
| Moisture | 3.861 | 0.045 | 0.001 | 0.006 |
| Sex | 2.293 | 0.027 | 0.003 | 0.013 |
| Group | 2.020 | 0.024 | 0.005 | 0.013 |
| BMI | 1.985 | 0.024 | 0.004 | 0.013 |
| Age | 1.760 | 0.021 | 0.019 | 0.041 |
| Calprotectin | 1.353 | 0.016 | 0.122 | 0.226 |
| GSRS | 1.135 | 0.014 | 0.276 | 0.448 |
| BDI | 0.974 | 0.012 | 0.475 | 0.685 |
| Time point | 0.446 | 0.011 | 1 | 1 |
| HAM-D | 0.742 | 0.009 | 0.847 | 1.000 |
| STAI1 | 0.649 | 0.008 | 0.933 | 1.000 |

*Notes.* R^2^ = explained variance; BH = Benjamini-Hochberg correction; GSRS = Gastrointestinal Symptom Rating Scale; BDI = Beck Depression Inventory; HAM-D = Hamilton Rating Scale for Depression; STAI1 = State-Trait Anxiety Inventory 1.

**Supplementary Table 8.** Changes in cortical thickness over time (ANOVA).

|  | Brain region | MNI | | | | | k | *F*_max_ | *p_peak (FWE)_* |  |
| --- | --- | --- | --- | --- | --- | --- | --- | --- | --- | --- |
|  |  | x | y | | z | |  |  |  | *p_cluster (FWE)_* |
| **Main effect Group** | | | | | | | | | | |
| **L** | Inferior temporal gyrus | -55 | | -37 | | -23 | 36 | 18.00 | 0.231 | 0.444 |
| **R** | Superior temporal gyrus | 54 | | -14 | | -9 | 112 | 17.03 | 0.310 | 0.032 |
| **L** | Superior frontal gyrus | -21 | | 31 | | 45 | 32 | 16.56 | 0.355 | 0.495 |
| **L** | Precentral gyrus | -55 | | -7 | | 20 | 75 | 16.03 | 0.411 | 0.124 |
| **R** | Superior temporal gyrus | 60 | | -39 | | 10 | 71 | 15.47 | 0.477 | 0.143 |
| **L** | Postcentral gyrus | -42 | | -29 | | 39 | 59 | 14.05 | 0.661 | 0.217 |
| **L** | Superior frontal gyrus | -21 | | 7 | | 50 | 17 | 13.80 | 0.694 | 0.702 |
| **L** | Superior temporal gyrus | -66 | | -38 | | 8 | 13 | 13.53 | 0.730 | 0.756 |
| **Main effect Time** | | | | | | | | | | |
| **R** | Parahippocampal gyrus | 21 | | -44 | | -11 | 7 | 12.90 | 0.807 | 0.830 |
| **Interaction Time*group** | | | | | | | | | | |
| **R** | Lateral orbitofrontal gyrus | 23 | | 30 | | -12 | 71 | 21.23 | 0.083 | 0.143 |
| **L** | Lingual gyrus | -5 | | -91 | | -5 | 10 | 13.17 | 0.775 | 0.794 |

*Notes*. R = right hemisphere; L = left hemisphere; *k =* cluster size in number of voxels; FWE = familywise error corrected.

**Supplementary Table 9.** Changes in gyrification over time (ANOVA).

|  | Brain region | MNI | | | | | k | *F*_max_ | *p_peak (FWE)_* |  |
| --- | --- | --- | --- | --- | --- | --- | --- | --- | --- | --- |
|  |  | x | y | | z | |  |  |  | *p_cluster (FWE)_* |
| **Main effect Group** | | | | | | | | | | |
| **L** | Middle frontal gyrus | -41 | | 10 | | 24 | 8 | 12.68 | 0.771 | 0.902 |
| **Main effect Time** | | | | | | | | | | |
| **L** | Inferior temporal gyrus | -48 | | -7 | | -33 | 10 | 14.44 | 0.793 | 0.870 |
| **L** | Insula | -39 | | -4 | | -15 | 10 | 14.33 | 0.806 | 0.870 |
| **Interaction Time*group** | | | | | | | | | | |
| **L** | Central sulcus | -39 | | -16 | | 35 | 28 | 14.93 | 0.733 | 0.474 |

*Notes*. L = left hemisphere; *k =* cluster size in number of voxels; FEW = familywise error corrected.

**Supplementary Table 10.** Changes in the sulcus depth over time (ANOVA).

|  | Brain region | MNI | | | | | k | *F*_max_ | *P_peak (FWE)_* |  |
| --- | --- | --- | --- | --- | --- | --- | --- | --- | --- | --- |
|  |  | x | y | | z | |  |  |  | *p_cluster (FWE)_* |
| **Main effect Group** | | | | | | | | | | |
| **L** | Medial superior frontal gyrus | -11 | | 42 | | 20 | 19 | 13.60 | 0.809 | 0.688 |
| **R** | Middle frontal gyrus | 26 | | 3 | | 50 | 24 | 13.67 | 0.802 | 0.596 |
| **L** | Middle frontal gyrus | -35 | | 23 | | 50 | 10 | 15.78 | 0.530 | 0.839 |
| **L** | Superior frontal gyrus | -21 | | -8 | | 61 | 15 | 14.64 | 0.679 | 0.759 |
| **R** | Supramarginal gyrus | 36 | | -42 | | 36 | 51 | 17.69 | 0.318 | 0.209 |
| **R** | Superior occipital gyrus | 13 | | -88 | | 35 | 29 | 19.55 | 0.181 | 0.506 |
| **Main effect Time** | | | | | | | | | | |
| *No suprathreshold clusters* | | | | | | | | | | |
| **Interaction Time*group** | | | | | | | | | | |
| *No suprathreshold clusters* | | | | | | | | | | |

*Notes*. R = right hemisphere; L = left hemisphere; *k =* cluster size in number of voxels; FEW = familywise error corrected.

**Supplementary Table 11.** Activation changes over time in face processing in the probiotics group.

|  | Brain region | MNI | | | | | | k | | *T*_max_ | | *P_peak(FWE)_* | |  |
| --- | --- | --- | --- | --- | --- | --- | --- | --- | --- | --- | --- | --- | --- | --- |
|  |  | x | | y | | z | |  |  |  |  |  |  | *P_cluster (FWE)_* |
| **Changes over time in neutral faces** | | | | | | | | | | | | | | |
| **Decreased activation** | | | | | | | | | | | | | | |
| **R** | Putamen, nucleus caudate | 20 | | 16 | | 10 | | 251 | | 4.78 | | | 0.246 | <0.001 |
| **L** | Putamen, nucleus caudate, pallidum | -18 | | 6 | | 12 | | 223 | | 4.06 | | | 0.782 | <0.001 |
| **Increased activation** | | | | | | | | | | | | | | |
| *No suprathreshold clusters* | | | | | | | | | | | | | | |
| **Changes over time in semi fearful faces** | | | | | | | | | | | | | | |
| *No suprathreshold clusters at all* | | | | | | | | | | | | | | |
| **Changes over time in fearful faces** | | | | | | | | | | | | | | |
| **Decreased Activation** | | | | | | | | | | | | | | |
| **L** | Middle occipital gyrus | | -20 | | -86 | | 12 | | 91 | | 3.96 | | 0.975 | 0.036 |
| **Increased activation** | | | | | | | | | | | | | | |
| *No suprathreshold clusters* | | | | | | | | | | | | | | |

*Notes*. R = right hemisphere; L = left hemisphere; *k =* cluster size in number of voxels; FWE = familywise error corrected.

**Supplementary Table 12.** Activation changes over time in face processing in the placebo group.

|  | Brain region | MNI | | | k | *T*_max_ | *P_peak (FWE)_* |  | |
| --- | --- | --- | --- | --- | --- | --- | --- | --- | --- |
|  |  | x | y | z |  |  |  | *P_cluster (FWE)_* | |
| **Changes over time in neutral faces** | | | | | | | | | |
| **Decreased activation** | | | | | | | | | |
| *No suprathreshold cluster* | | | | | | | | | |
| **Increased activation** | | | | | | | | | |
| **R** | Cuneus, calcarine gyrus | 8 | -82 | 30 | 203 | 5.00 | 0.126 | <0.001 | |
| **Changes over time in semi fearful faces** | | | | | | | | | |
| **Decreased activation** | | | | | | | | | |
| **R** | Inferior frontal gyrus | 48 | 10 | 26 | 122 | 4.53 | 0.485 | 0.009 | |
| **L** | Inferior frontal gyrus | -42 | 20 | 24 | 105 | 5.00 | 0.126 | 0.019 | |
| **R** | Middle occipital gyrus, angular gyrus | 30 | -68 | 34 | 202 | 4.34 | 0.595 | <0.001 | |
| **Increased Activation** | | | | | | | | | |
| **L** | Superior frontal gyrus, anterior cingulum | -10 | 56 | 16 | 98 | 4.05 | 0.268 | 0.026 | |
| **R** | Cuneus, superior occipital gyrus | 10 | -82 | 30 | 85 | 4.48 | 0.942 | 0.048 | |
| **Changes over time in fearful faces** | | | | | | | | | |
| **Decreased Activation** | | | | | | | | | |
| *No suprathreshold clusters* | | | | | | | | | |
| **Increased activation** | | | | | | | | |  |
| **L** | Middle cingular cortex | -6 | -26 | 32 | 199 | 5.05 | 0.105 | <0.001 | |
| **R** | Cuneus, calcarine sulcus | 10 | -82 | 30 | 211 | 5.45 | 0.026 | <0.001 | |

*Notes*. R = right hemisphere; L = left hemisphere; *k =* cluster size in number of voxels; FWE = familywise error corrected.

**Supplementary Table 13.** **Patients versus healthy controls during neutral face processing.**

|  | Brain region | MNI | | | k | *T*_max_ | *P_peak (FWE)_* |  |
| --- | --- | --- | --- | --- | --- | --- | --- | --- |
|  |  | x | y | z |  |  |  | *P_cluster (FWE)_* |
| **Patients > Healthy controls** | | | | | | | | |
| **B** | Medial superior frontal gyrus, anterior cingulate gyrus | 4 | 28 | 42 | 74 | 7.14 | <0.001 | <0.001 |
| **R** | Amygdala, putamen | 22 | 4 | -10 | 21 | 6.31 | <0.001 | <0.001 |
| **L** | Putamen, nucleus caudate | -18 | 2 | 12 | 31 | 7.07 | <0.001 | <0.001 |
| **L** | Rolandic operculum, insula | -36 | -6 | 16 | 17 | 6.53 | <0.001 | <0.001 |
| **L** | Supramarginal gyrus | -58 | -22 | 40 | 15 | 6.07 | <0.001 | <0.001 |
| **L** | Cerebellum, fusiform gyrus | -34 | -42 | -24 | 25 | 6.34 | <0.001 | <0.001 |
| **R** | Fusiform gyrus | 46 | -62 | -28 | 19 | 6.73 | <0.001 | <0.001 |
| **R** | Fusiform gyrus, cerebellum | 26 | -82 | -16 | 17 | 6.43 | <0.001 | <0.001 |
|  |  | 38 | -52 | -22 | 15 | 6.09 | <0.001 | <0.001 |
| **R** | Cerebellum superior posterior lobe | 26 | -56 | -20 | 27 | 6.70 | <0.001 | <0.001 |
|  |  | 34 | -70 | -20 | 33 | 6.51 | <0.001 | <0.001 |
| **L** | Cerebellum superior posterior lobe | -32 | -60 | -30 | 171 | 7.42 | <0.001 | <0.001 |
| **L** | Cerebellum | -6 | -80 | -32 | 30 | 6.35 | <0.001 | <0.001 |
| **Healthy controls > Patients** | | | | | | | | |
| **L** | Precentral gyrus | -28 | -22 | 62 | 71 | 8.23 | <0.001 | <0.001 |
| **R** | Lingual gyrus | 10 | -76 | -2 | 72 | 7.56 | <0.001 | <0.001 |
| **R** | Calcarine sulcus | 12 | -88 | 12 | 17 | 6.58 | <0.001 | <0.001 |

*Notes*. R = right hemisphere; L = left hemisphere; B = bilateral; *k =* cluster size in number of voxels; FEW = familywise error corrected.

**Supplementary Table 14.** Patients versus healthy controls during semi-fearful face processing.

|  | Brain region | | MNI | | | k | *T*_max_ | *P_peak (FWE)_* |  |
| --- | --- | --- | --- | --- | --- | --- | --- | --- | --- |
|  |  |  | x | y | z |  |  |  | *P_cluster (FWE)_* |
| **Patients > Healthy Controls** | | | | | | | | | |
| **L** | | Rolandic operculum | -40 | -8 | 16 | 11 | 5.93 | 0.015 | 0.001 |
| **Healthy controls > Patients** | | | | | | | | | |
| **R** | | Precentral gyrus | 16 | -26 | 56 | 65 | 6.76 | 0.001 | <0.001 |
| **L** | | Precentral gyrus | -28 | -24 | 56 | 284 | 9.20 | <0.001 | 0.026 |
| **L** | | Middle cingulate gyrus, paracentral lobule | -8 | -30 | 50 | 26 | 7.14 | <0.001 | <0.001 |
| **R** | | Lingual gyrus, calcarine gyrus | 8 | -78 | 0 | 168 | 9.47 | <0.001 | <0.001 |
| **L** | | Lingual gyrus | -8 | -76 | -4 | 29 | 6.53 | 0.002 | <0.001 |
| **R** | | Superior occipital gyrus | 24 | -80 | 24 | 14 | 6.06 | 0.01 | <0.001 |
| **R** | | Calcarine gyrus, cuneus | 8 | -90 | 8 | 43 | 7.19 | <0.001 | 0.026 |
| **L** | | Cuneus, superior occipital gyrus | -8 | -96 | 16 | 29 | 6.12 | 0.008 | <0.001 |

*Notes*. R = right hemisphere; L = left hemisphere; *k =* cluster size in number of voxels; FWE = familywise error corrected.

**Supplementary Table 15.** Patients versus healthy controls during fearful faces processing.

|  | Brain region | | MNI | | | | | | k | | *T*_max_ | | *P_peak (FWE)_* | |  | |  |
| --- | --- | --- | --- | --- | --- | --- | --- | --- | --- | --- | --- | --- | --- | --- | --- | --- | --- |
|  |  |  | x | | y | | z | |  |  |  |  |  |  | *P_cluster (FWE)_* | |  |
| **Patients > Healthy Controls** | | | | | | | | | | | | | | | | | |
| **L** | | Cerebellum, fusiform gyrus | | -40 | | -54 | | -22 | | 26 | | 6.23 | | 0.006 | | <0.001 | |
| **Healthy controls > Patients** | | | | | | | | | | | | | | | | | |
| **L** | | Precentral gyrus | | -28 | | -22 | | 62 | | 77 | | 7.28 | | <0.001 | | <0.001 | |
| **R** | | Lingual gyrus | | 10 | | -78 | | -2 | | 52 | | 7.37 | | <0.001 | | <0.001 | |
| **R** | | Calcarine gyrus | | 10 | | -90 | | 10 | | 12 | | 6.12 | | <0.001 | | <0.001 | |

*Notes*. R = right hemisphere; L = left hemisphere; *k =* cluster size in number of voxels; FWE = familywise error corrected.

**Supplementary References**

1. World Health Organization. WHO Collaborating Centre for Drug Statistics Methodology: ATC classification index with DDDs, 2020. 2019.

2. Gupta SK. Intention-to-treat concept: a review. *Perspectives in clinical research* 2011; **2**(3)**:** 109.

3. Cramer JA, Rosenheck R. Compliance with medication regimens for mental and physical disorders. *Psychiatric Services* 1998; **49**(2)**:** 196-201.

4. Kowarik A, Templ M. Imputation with the R Package VIM. *Journal of Statistical Software* 2016; **74**(7)**:** 16.

5. Bates D, Machler M, Bolker BM, Walker SC. Fitting Linear Mixed-Effects Models Using lme4. *Journal of Statistical Software* 2015; **67**(1)**:** 1-48.

6. Riedel M, Moller HJ, Obermeier M, Schennach-Wolff R, Bauer M, Adli M *et al.* Response and remission criteria in major depression - A validation of current practice. *Journal of Psychiatric Research* 2010; **44**(15)**:** 1063-1068.

7. Falony G, Joossens M, Vieira-Silva S, Wang J, Darzi Y, Faust K *et al.* Population-level analysis of gut microbiome variation. *Science* 2016; **352**(6285)**:** 560-564.

8. Callahan BJ, McMurdie PJ, Rosen MJ, Han AW, Johnson AJA, Holmes SP. DADA2: High-resolution sample inference from Illumina amplicon data. *Nature Methods* 2016; **13**(7)**:** 581-+.

9. Vandeputte D, Kathagen G, D'Hoe K, Vieira-Silva S, Valles-Colomer M, Sabino J *et al.* Quantitative microbiome profiling links gut community variation to microbial load. *Nature* 2017; **551**(7681)**:** 507-+.

10. Prest EI, Hammes F, Kötzsch S, van Loosdrecht MC, Vrouwenvelder JS. Monitoring microbiological changes in drinking water systems using a fast and reproducible flow cytometric method. *Water Res* 2013; **47**(19)**:** 7131-7142.

11. Kurilshikov A, Medina-Gomez C, Bacigalupe R, Radjabzadeh D, Wang J, Demirkan A *et al.* Large-scale association analyses identify host factors influencing human gut microbiome composition. *Nature Genetics* 2021; **53**(2)**:** 156-+.

12. Zhang XY, Yi NJ. NBZIMM: negative binomial and zero-inflated mixed models, with application to microbiome/metagenomics data analysis. *Bmc Bioinformatics* 2020; **21**(1).

13. Mugler III JP, Brookeman JR. Three‐dimensional magnetization‐prepared rapid gradient‐echo imaging (3D MP RAGE). *Magnetic resonance in medicine* 1990; **15**(1)**:** 152-157.

14. Gaser C, Dahnke R. CAT-a computational anatomy toolbox for the analysis of structural MRI data. *HBM* 2016; **2016:** 336-348.

15. Filkowski MM, Haas BW. Rethinking the Use of Neutral Faces as a Baseline in fMRI Neuroimaging Studies of Axis-I Psychiatric Disorders. *Journal of Neuroimaging* 2017; **27**(3)**:** 281-291.
